# Profiling genes encoding the adaptive immune receptor repertoire with gAIRR Suite

**DOI:** 10.3389/fimmu.2022.922513

**Published:** 2022-09-08

**Authors:** Mao-Jan Lin, Yu-Chun Lin, Nae-Chyun Chen, Allen Chilun Luo, Sheng-Kai Lai, Chia-Lang Hsu, Jacob Shujui Hsu, Chien-Yu Chen, Wei-Shiung Yang, Pei-Lung Chen

**Affiliations:** ^1^ Department of Medical Genetics, National Taiwan University Hospital, Taipei, Taiwan; ^2^ Department of Computer Science, Johns Hopkins University, Baltimore, MD, United States; ^3^ Graduate Institute of Medical Genomics and Proteomics, National Taiwan University, Taipei, Taiwan; ^4^ Academia Sinica and National Taiwan University, Taipei, Taiwan; ^5^ Graduate Institute of Oncology, School of Medicine, National Taiwan University, Taipei, Taiwan; ^6^ Department of Medical Research, National Taiwan University Hospital, Taipei, Taiwan; ^7^ Department of Biomechatronics Engineering, National Taiwan University, Taipei, Taiwan; ^8^ Division of Endocrinology and Metabolism, Department of Internal Medicine, National Taiwan University Hospital, Taipei, Taiwan; ^9^ Graduate Institute of Clinical Medicine, College of Medicine, National Taiwan University, Taipei, Taiwan

**Keywords:** adaptive immune receptor repertoire (AIRR), allele typing, targeted sequencing, immunogenomics, novel allele, germline genes encoding AIRR (gAIRR)

## Abstract

Adaptive immune receptor repertoire (AIRR) is encoded by T cell receptor (TR) and immunoglobulin (IG) genes. Profiling these germline genes encoding AIRR (abbreviated as gAIRR) is important in understanding adaptive immune responses but is challenging due to the high genetic complexity. Our gAIRR Suite comprises three modules. gAIRR-seq, a probe capture-based targeted sequencing pipeline, profiles gAIRR from individual DNA samples. gAIRR-call and gAIRR-annotate call alleles from gAIRR-seq reads and annotate whole-genome assemblies, respectively. We gAIRR-seqed TRV and TRJ of seven Genome in a Bottle (GIAB) DNA samples with 100% accuracy and discovered novel alleles. We also gAIRR-seqed and gAIRR-called the TR and IG genes of a subject from both the peripheral blood mononuclear cells (PBMC) and oral mucosal cells. The calling results from these two cell types have a high concordance (99% for all known gAIRR alleles). We gAIRR-annotated 36 genomes to unearth 325 novel TRV alleles and 29 novel TRJ alleles. We could further profile the flanking sequences, including the recombination signal sequence (RSS). We validated two structural variants for HG002 and uncovered substantial differences of gAIRR genes in references GRCh37 and GRCh38. gAIRR Suite serves as a resource to sequence, analyze, and validate germline TR and IG genes to study various immune-related phenotypes.

## 1 Introduction

Adaptive immune receptor repertoire (AIRR) includes B cell receptors, or immunoglobulin (IG), and T cell receptors (TR) and reflects antigen-specific immune responses. The receptors’ final sequences require somatic recombination, where one gene from each variable (V), diversity (D) and joining (J) genetic region are selected and rearranged into a continuous fragment (1, 2), with or without additional somatic hypermutation. D gene segments are only present in the heavy chain locus of IG and the beta and delta chain loci of TR. Humans’ immune repertoire has huge variability capable of binding all kinds of antigens (3–5), but for each individual the number of V(D)J genes/alleles is limited (3). Studies have shown that the germline IG and TR compositions can affect immunological responses (3, 6, 7). Early data suggested SARS-CoV-2 antibodies prefer to use certain IG genes/alleles (8, 9). Carbamazepine-induced Stevens-Johnson syndrome (10), besides the well-known HLA-B*15:02 susceptibility in Asians, also depends on specific public TRs for the severe cutaneous adverse reaction (11). In a simian immunodeficiency virus infection model, the TR alleles responsible for the potent CD8+ T cell response were recently determined (12). Germline TR and IG alleles have started to be found associated with human diseases, including rheumatic heart disease (13), and Kawasaki disease (14, 15). These examples are likely only the tip of the iceberg because of the complexity in determining individual germline variation in TR and IG genes and the lack of diverse and comprehensive AIRR database (16). The international ImMunoGeneTics (IMGT) database (5), the central depository of germline TR and IG genes, is a valuable resource but has been slow in adding new alleles.

AIRR profiling methods can use messenger RNA (mRNA)/complementary DNA (cDNA) or genomic DNA (gDNA) data, albeit they yield different information (16, 17). The popular mRNA/cDNA approach provides useful dynamic information of AIRR clonotypes and expression levels, which can be valuable if, and only if, the most relevant lymphocytes can be retrieved from the right site at the right time (16). mRNA/cDNA-based AIRR sequencing at any given time point can only cover part of the full spectrum of AIRR. Depending on the library preparation strategies, the mRNA/cDNA approach might suffer from allele dropout, lack of full-length coverage and inconsistent results (18). Furthermore, the non-coding flanking sequences, including RSS which may significantly affect recombination efficiency, cannot be profiled in the mRNA/cDNA approach. On the other hand, bulk gDNA includes non-rearranged and rearranged V(D)J sequences. Non-rearranged gDNA shows the germline genetic information of an individual. Rearranged V(D)J sequences reflects the state between germline and transcribed sequences (16). gDNA is more stable and easier for storage compared to mRNA, with millions of gDNA samples already stored in the clinical setting or in previous genetic studies. Although mRNA/cDNA-based AIRR can be profiled by many methods, gDNA-based gAIRR has just started to attract attention (19–21).

Computational methods have been designed to profile AIRR and germline TR and IG genes. IgDiscover (22), TIgGER (23), RAbHIT (24), and TCR_Genotype (25) analyzed mRNA or somatically V(D)J-recombined gDNA data (AIRR-seq) to profile TR or IG genes. IGenotyper (19) uses targeted gDNA long-read sequencing to profile germline IGHV genes. ImmunoTyper-SR (26) uses whole-genome sequencing (WGS) gDNA short-read data to profile germline IGHV genes. While these gDNA-based tools provide comprehensive information for germline TR and IG gene profiling, the long-read-based method is cost intensive and the accuracy of the WGS-based method is limited by the sequencing coverage.

**Figure 1:**
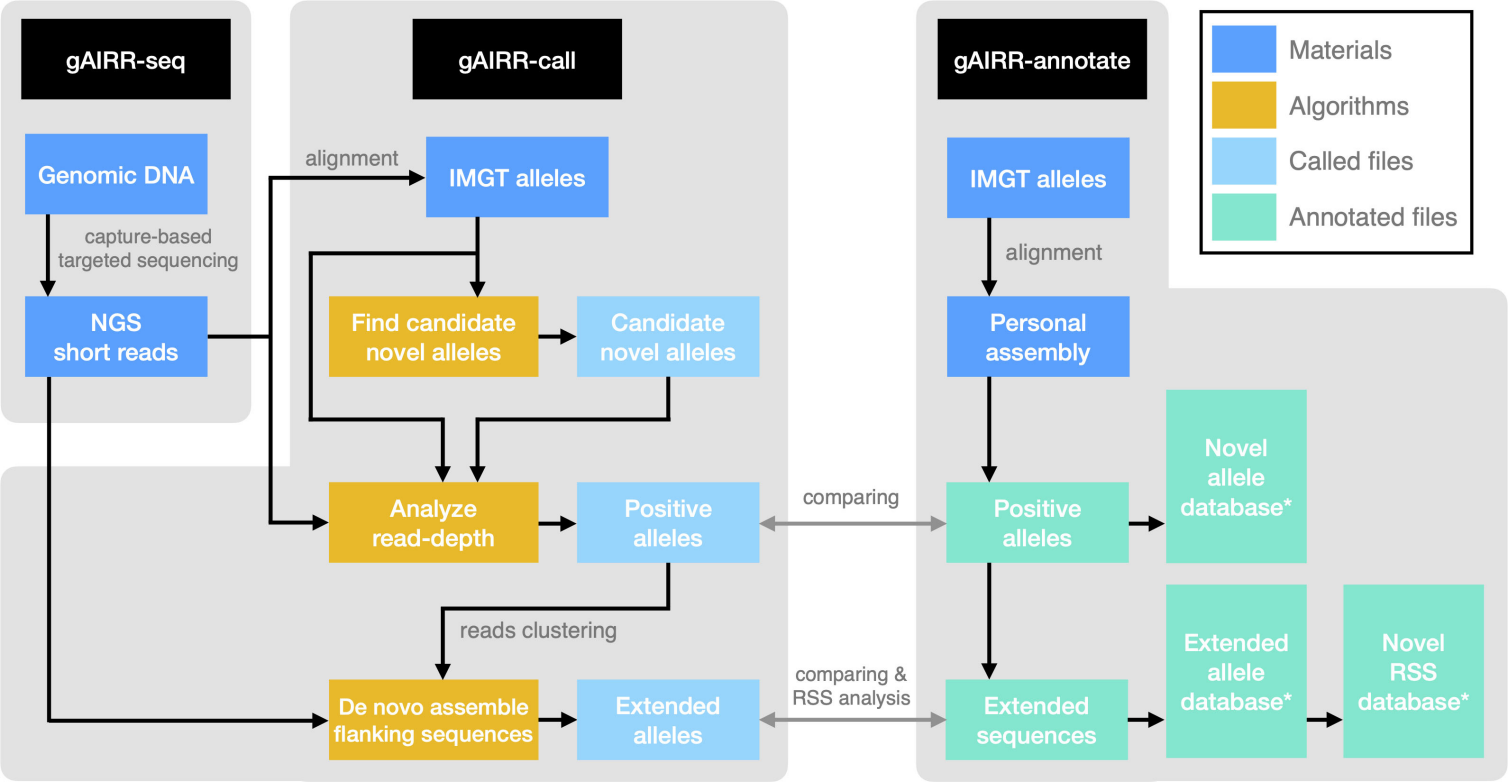
The gAIRR Suite pipelines. The gray arrows show the verification methods between the two pipelines when both gAIRR-seq reads and personal assembly are available. (Section 2.5). *: These database can also be generated by gAIRRseq + gAIRR-call.

In this study, we developed the gAIRR Suite comprising three modules (**Figure 1**). gAIRR-seq, a probe capture-based targeted gDNA sequencing pipeline, profiles germline TR and IG genes from individual DNA samples. The probe hybridization method can tolerate sequence mismatch to a certain degree and therefore can discover novel alleles. gAIRR-call (**Figure 1**), using a coarse-to-fine strategy, starts from aligning gAIRR-seq reads to known AIRR alleles collected from the IMGT database (5), to eventually call both known and novel AIRR alleles, as well as their flanking sequences. One major challenge for germline TR and IG studies is the lack of curated annotations and no ground truth for tool development. We designed gAIRR-annotate to annotate gAIRR alleles and flanking sequences, including novel ones, using whole-genome assemblies (**Figure 1**). In this study we discovered numerous novel alleles, structural variants, substantial differences in different versions of the reference genome, and RSS polymorphism across subjects. The gAIRR Suite can potentially benefit future genetic study and clinical applications for various immune-related phenotypes.

## 2 Methods

### 2.1 The gAIRR-seq pipeline: a capture-based targeted sequencing method

#### 2.1.1 Reference materials

We obtained 7 GIAB genomic DNA reference materials (RMs) from the Coriell Institute (https://www.coriell.org). These 7 RMs included a pilot genome HG001/NA12878 and two Personal Genome Project trios - an Ashkenazim Jewish ancestry: HG002/huAA53E0/NA24385, HG003/hu6E4515/NA24149, HG004/hu8E87A9/NA24143; a Chinese ancestry: HG005/hu91BD69/NA24631, HG006/huCA017E/NA24694, HG007/hu38168C/NA24695. The genomic DNA samples were retrieved from EBV-transformed B lymphocytes. These RMs were well-sequenced using more than 10 NGS platforms/protocols by GIAB (27); therefore, they are appropriate RMs for benchmarking gAIRR-seq.

#### 2.1.2 Primary cell samples

We also extracted the genomic DNA from both the peripheral blood mononuclear cells (PBMC) and oral mucosal cells of a Taiwanese subject. The subject signed informed consent for participating in the project, and all procedures were approved by the Research Ethics Committee of the National Taiwan University Hospital.

#### 2.1.3 Probe design

We designed probes for all known V and J alleles of IG and TR based on the IMGT database Version 3.1.22 (3 April 2019) (including all functional, ORF, and pseudogenes) (5). Each probe was a continuous 60-bp oligo (Roche NimbleGen, Madison, WI, U.S.). We designed three probes for each V allele and one probe for each J allele based on V and J alleles’ length differences. (**Supplementary Figure S16**). For J alleles shorter than 60 bp, we padded the probes with random nucleotides ‘N’ to 60 bp in length on both ends. An example of probes and captured reads’ relative position is shown in **Supplementary Section 19**.

#### 2.1.4 Library preparation

We quantified the input of 1000 ng gDNA and assessed the gDNA quality using a Qubit 2.0 fluorometer (Thermo Fisher Scientific, Waltham, MA, U.S.) before the library preparation. The gDNA was fragmented using Covaris (Coravis, Woburn, MA, U.S.), aiming at the peak length of 800 bp, which was assessed using Agilent Bioanalyzer 2100 (Agilent Technologies, Santa Clara, CA, U.S.). We then added adapters and barcodes onto the ends of the fragmented DNA to generate indexed sequencing libraries using TruSeq Library Preparation Kit (Illumina, San Diego, CA, U.S.). We performed capture-based target enrichment using the SeqCap EZ Hybridization and Wash Kit (Roche NimbleGen, Madison, WI, U.S.). We then used the sequencing platform, MiSeq (Illumina, San Diego, CA, U.S.), to generate paired-end reads of 300 nucleotides.

### 2.2 The gAIRR-call pipeline

The gAIRR-call pipeline is composed of three steps: Finding novel alleles, calling alleles, and generating extended alleles with 200 bp flanking sequences on both ends.

#### 2.2.1 Finding novel allele candidates

gAIRR-call first generated candidate novel alleles using capture-based short reads. The capture-based short reads were aligned to all the known alleles in the IMGT database (v3.1.22 in this study). Then gAIRR-call checked the reference IMGT alleles one by one. For any known allele, the variants were called with the aligned reads. gAIRR-call marked any allele position with more than a quarter of reads supporting bases different from the reference. If there was only one variant in the reference, gAIRR-call simply called the allele with the variant as a novel allele candidate. If there is more than one variant in the reference, gAIRR-call performs phasing and connects the variants with aligned reads. Since most of the AIRR alleles were less than 300 bp in length, there were usually a sufficient number of captured reads (2 x 300 bp in length using gAIRR-seq) for phasing.

gAIRR-call then collected called haplotypes and checked if there were duplicated sequences. If a called haplotype was actually another known allele in IMGT, the haplotype was discarded. Because haplotypes were called from known alleles with different lengths, there could be duplicated haplotypes representing the same allele. In this case, only the shorter haplotype was kept. After de-duplicating and cleaning, the remaining haplotypes were unique. The haplotypes joined the known IMGT alleles to be the candidate alleles for the next stage analysis.

#### 2.2.2 Calling AIRR alleles

Compared to the previous step, calling AIRR allele applied stricter criteria. gAIRR-call aligned capture-based short reads to the allele candidates pool, which included both known IMGT alleles and the novel ones. To ensure that every candidate allele could be aligned by all potential matched reads, we used the ‘-a’ option of BWA MEM (28) so that one read could be aligned to multiple positions.

**Figure 2:**
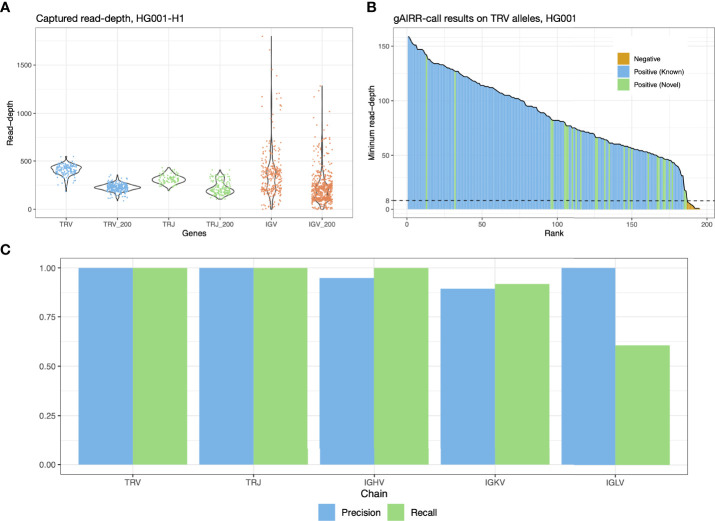
gAIRR-seq and gAIRR-call results. **(A)** Read depths sequenced with gAIRR-seq in TRV, TRJ and IGV regions using data from HG001. Columns without the “_200” suffix shows the average read-depth of a region. Columns with the “_200” suffix shows the read depth 200 bp away from the region boundaries. **(B)** gAIRR-call results using HG001 data. The results are sorted by minimum read-depth of the perfect matched reads. The dash line represents the adaptive threshold in gAIRR-call. All the alleles not annotated by gAIRR-annotate, colored in orange, are below the adaptive threshold and are regarded as nocalls by gAIRR-call. True known alleles are in blue and true novel alleles are in green. In the HG001 analysis all true alleles are successfully identified by gAIRR-call. Alleles with zero minimum read-depth are not included in the figure. **(C)** gAIRR-call concordance with manual inspected gAIRR-annotate results for HG001. For IGHV, IGKV, and IGLV regions, we only included the functional genes.

We only considered reads perfectly matched and sharing a long overlap with the allele. For alleles longer than 100 bp, we required 100-bp exact overlap. For shorter ones, we required perfect overlap. The stringent filtering removed false positive read assignments. gAIRR-call counted the remaining reads’ read-depth of all allele positions. The minimum read-depth in an allele was its final score relative to other candidate alleles. The candidate alleles were sorted according to their supporting minimum read-depth (**Figure 2B**).

We used an adaptive threshold in gAIRR-call to decide the final calls. The goal of this adaptive threshold was to identify the large drop in minimum read-depth, which was observed to be a good indicator of true-positive and false-positive alleles. When two consecutive alleles had a read depth ratio of less than 0.75, we set the adaptive threshold to be the lower read depth. All the candidate alleles with lower minimum read-depth below the adaptive threshold were discarded. We also noticed that there could be some alleles with extraordinary allele lengths. For example, *TRAV8-5* is a 1355 bp pseudogene that is the longest known TR alleles, where the second-longest allele is 352 bp. When they truly appeared in the data, their minimum read-depths were usually slightly lower than other true alleles’. Thus, when calculating the adaptive threshold, we excluded outlier alleles like *TRAV8-5* to provide a more appropriate threshold. After that, we still compared the minimum read-depth of these outlier alleles with the adaptive threshold to decide whether they were positive.

#### 2.2.3 Calling flanking sequences

In addition to allele calling, gAIRR-call reports flanking sequences of an allele when possible. We included the mate of the supporting reads of each allele for flanking sequences calling. We then assembled the reads using SPAdes (v3.11.1) (29) with the ‘–only-assembler’ option. We discarded contigs not containing the exact sequences of an allele. Usually an allele generated one contig. We kept the longest contig if multiple were generated for one allele.

We phased the locally assembled contigs to obtain accurate flanking sequences for both haplotypes (H1 and H2) of a sample. We realigned the captured reads to all local contigs and took the primary alignment. Here we assumed that the flanking sequences of an allele in the two haplotypes were similar and thus the reads of an allele’s two flanking sequences could be aligned to the same contig. We marked the contig position with more than a quarter of reads supporting bases different from the contig. Then we phased the variants using the pair-end reads. The fragment lengths of the pair-end reads were up to 800 bp; hence the allele region and extended 200 bp from both sides can be fully covered by the pair-end reads. After phasing, the extended alleles with 200 bp flanking sequences were reported.

Although the contigs assembled by SPAdes were typically longer than 1000 bp, we did not report the full-length flanking sequences for two reasons. First, the contigs’ boundary regions were not very reliable because the read depth was decreasing toward the boundaries. Second, the fragment length of our capture-based reads was up to 800 bp. If the shortest distance between variants on two flanking sides of the allele is longer than 800 bp, it would not be possible to phase the variants. Considering most AIRR alleles are shorter than 300 bp, it is robust to call the extended alleles with 200 bp flanking sequences from both sides.

We performed flanking sequence calling after completion of novel alleles calling for the following reason. If the reads can only be aligned to contigs generated by known alleles, the reads of novel alleles will be aligned to the closest known alleles, resulting in false-positive read assignment and lower accuracy.

### 2.3 The gAIRR-annotate pipeline

The gAIRR-annotate pipeline annotated AIRR alleles as well as the flanking sequences using publicly available whole-genome assemblies (**Supplementary Table S21**). gAIRR-annotate begined with aligning IMGT AIRR alleles to the assemblies. To take novel alleles into consideration, we set the alignment options to allow mismatches and indels, and reported all potential sites. For any allele aligned with mismatches or indels, we identified its nearest allele in the IMGT database and assigned a novel allele to the associated gene. When there were multiple assemblies available for the same sample, we only annotated an allele if more than half of the assemblies reported the same output.

To find RSS of each allele, we aligned all IMGT RSS to the annotated flanking sequences. For an RSS aligned with mismatch(es), we defined the aligned region as a novel RSS. The flanking sequences unable to be aligned with IMGT RSS were re-aligned where IMGT heptamers and nonamers were separated. The second-pass processing thus allowed heptamer-nonamer pairs not recorded in IMGT (**Supplementary Figure S19**).

For TRV and TRJ alleles, gAIRR-annotate used the allelic sequences as the query. For TRD alleles, because the lengths of them range from 8 to 16 bp, which would result in a huge number of valid alignments, we extended them by adding the heptamers on both sides of the D region. After the extension with the heptamers, the extended D alleles’ length ranged from 21 to 31. They usually appeared only once in the whole-genome, suggesting the uniqueness of TRD alleles with appropriate RSS.

### 2.4 HQ-12 set

The HQ-12 set was selected where each sample could either be validated with family information or was curated with additional effort. This dataset includes HG001 (also known as NA12878, Northwest European American from Utah), HG002 (also known as NA24385, Ashkenazi Jewish), Puerto Rican trio (HG00731, HG00732, and HG00733), Southern Han Chinese trio (HG00512, HG00513, and HG00514), Yoruban trio (NA19238, NA19239, and NA19240), and a haploid hydatidiform mole CHM13. All samples have multiple assemblies generated from different technologies, assembly software or research groups.

The alleles called using different assemblies from a single sample were concordant in most cases. The pipeline used a conservative strategy for high-precision annotation at conflicted loci – it took the alleles called from more than a half of the assemblies at a locus when the assemblies of a single sample generated inconsistent results. For example, we included six HG002 assemblies in the analysis, so gAIRR-annotate took an allele when there were calls from at least four assemblies. (**Supplementary Table S22**).

### 2.5 Comparing alleles of gAIRR-call and gAIRR-annotate

Because both the capture-based sequence data and personal assembly were available in HG001 and HG002, we could compare the results of gAIRR-call and gAIRR-annotate in these two samples. The known alleles identified by gAIRR-call could be directly compared with gAIRR-annotate results; however, comparing the novel alleles were not as straightforward as comparing known ones. As a result, we verified the gAIRR-call novel alleles by aligning them to the personal assemblies. If a novel allele could perfectly align to the assembly, then we considered it as a true positive. We also checked if gAIRR-call alleles covered all the annotated novel allele positions in gAIRR-annotate. Similarly, since there were novel alleles in extended alleles, we evaluated the flanking sequences with alignment rather than direct comparisons. If an annotation is not called by gAIRR-call, it’s regarded as false-negative; if a gAIRR-call result doesn’t have a matched annotation, it’s considered to be false-positive.

### 2.6 Verifying HG002’s deletion with gAIRR-seq reads

We aligned capture-based short reads of gAIRR-seq from HG002. We first examined if there were reads aligned across two deletion breakpoints, either in a chimeric form or having two paired segments separated across the region. We found 72 reads aligned across chr14:22,918,113 and chr14:22,982,924 using the gAIRR-seq dataset, providing clear evidence of a long deletion.

We also compared the alignment result of HG002 to those of HG003 (HG002’s father) and HG004 (HG002’s mother). We compared the read depth inside and outside the called deletion region. We counted the number of reads in the centromeric and the telomeric regions of chr14:22,982,924 and calculated the coverage drop-off rate (#centromeric reads/#telomeric reads). In a region where reads are nearly uniformly aligned, the drop-off rate is expected to be around 1. If one of the haplotypes is missing on the centromeric side, we expect a drop-off rate around 0.5. At locus chr14:22,982,924, the drop-off rate of HG002 is 0.622, while the values for HG003 and HG004 are 1.005 and 1.138 respectively (**Supplementary Table S24**). This verified the long deletion in the HG002 TRA/TRD region and further provided evidence that the structural variant was likely a novel mutation.

### 2.7 Database collecting

We deposited the novel alleles and the extended alleles with 200 bp flanking sequences called by gAIRR-annotate into two databases. Usually, the alleles called by gAIRR-annotate using different assemblies from a single sample are concordant. When there are conflicts between different assemblies, the pipeline uses a conservative strategy for high precision - it takes the alleles called from more than a half of the assemblies at a locus when the assemblies of a single sample generated inconsistent results. For example, we included six HG002 assemblies in the analysis, so gAIRR-annotate took an allele when there were calls from at least four assemblies. We also collected novel alleles and extended alleles called by gAIRR-call. Whenever there are multiple forms of the same allele called, which are inferred from different ways, we keep only the shortest one.

## 3 Results

### 3.1 gAIRR-seq achieved efficient targeted sequencing in TR and IG regions

We gAIRR-seqed GIAB RMs HG001-7 (EBV-transformed B lymphocytes) and two primary cell samples (PBMC and oral mucosal cells from the same individual) to generate paired-end reads (2 x 300 bp), with a mean of 310 thousand reads for each DNA sample. To evaluate the sequencing quality and efficiency, we aligned the gAIRR-seq reads of the GIAB RMs, HG001 and HG002, to their individual whole-genome assemblies (30), with over 96% of the reads successfully aligned. Over 83% of the sequenced reads were on-target in the AIRR regions in HG001 and HG002 (**Supplementary Table S1**). In TRV and TRJ allelic regions where probes were designed, the average read depth was above 350; the sequencing depth at positions 200 bp away from the allele boundaries was typically above 200x (**Figure 2A**). Much higher read depth variation was found in IGV alleles, which might be biased because the DNA was from EBV-transformed B lymphocytes (see Discussion). gAIRR-seq efficiently enriches gAIRR sequences with high on-target rate and high coverage and can provide high-quality data for analyses.

### 3.2 AIRR-call could profile known and novel germline TRV, TRJ, and IGV alleles

We applied gAIRR-call using the high-coverage targeted reads from gAIRR-seq. We collected known alleles in the IMGT database and used them to identify candidate alleles. We define an allele call to be positive if the candidate allele has higher read coverage than a dataset-dependent adaptive threshold (Section 2.2).

To validate the accuracy of the gAIRR-call results, we compared them with the high-confidence TR annotations from gAIRR-annotate on HG001 and HG002 using their phased whole-genome assemblies (Section 3.3). gAIRR-call results were 100% concordant with the annotations in TRV and TRJ regions for both known and novel alleles (**Figures 2B, C**). We then evaluated the performance of gAIRR-call in functional IGV genes for HG001 (**Figure 2C**; see Section 3.3 for validation details). gAIRR-call had 100.0% recall (true positive rate) and 94.9% precision (positive predictive value) for the functional IGHV genes when comparing to our annotations. The precision of gAIRR-call was 89.5% for IGKV and 100.0% for IGLV, and the recall was 91.9% for IGKV but 60.5% for IGLV. We examined the gAIRR-seq data in IGLV and observed substantial coverage drop, suggesting many sequenced cells had been somatically V(D)J-recombined (**Supplementary Figure S2A**).

**Figure 3:**
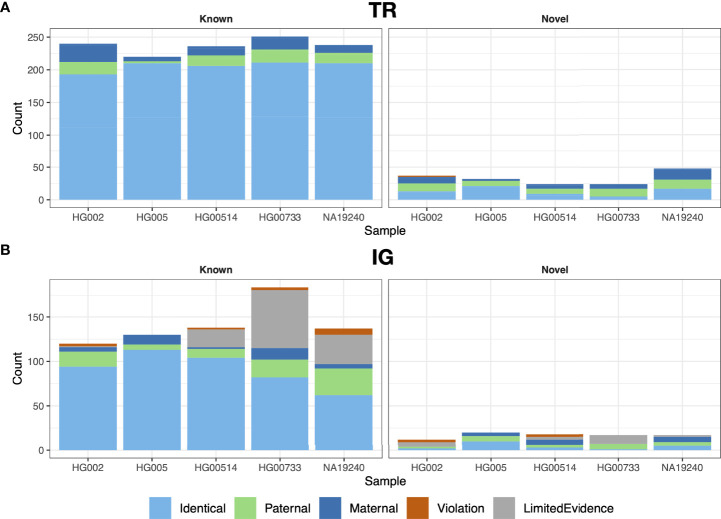
Trio validation for gAIRR-call and gAIRR-annotate. We validated gAIRR-call using the HG002 and HG005 families and validated gAIRR-annotate using the HG00514, HG00733, and NA19240 families. **(A)** TR loci, including all genes in TRV and TRJ. **(B)** IG loci, including functional IGV genes. The genes with V(D)J recombination evidence and genes without parental information due to gene lost are classified as LimitedEvidence.

We performed trio analysis for gAIRR-call results using an Ashkenazi family (son: HG002, father: HG003 and mother: HG004) and a Chinese family (son: HG005, father: HG006 and mother: HG007; **Figure 3**). There were no Mendelian violations observed in either family in TR regions except for two HG002 genes (*TRAJ29* and *TRBJ2-3*) (**Figure 3A** and **Supplementary Figure S1**). We inspected the violated alleles and noticed they both located near known structural variant breakpoints, which were both regarded as *de novo* mutations (Section 3.5). In IG regions, gAIRR-call results had very low Mendelian violation rates for known alleles (2.5% in IGHV for HG002 and 0% otherwise; **Figure 3B**). Mendelian violation rates were higher for novel alleles. We manually inspected the annotated novel alleles and 3 of them had direct evidence of V(D)J recombination (*IGHV3-48*, *IGKV3-20*, *IGLV2-14*; see **Supplementary Figure S3**). We also observed gAIRR-seq coverage drops in the parents’ samples, which could also result from somatic V(D)J-recombination (**Supplementary Figure S4**). We additionally validated 12 TRV and 9 IGV novel alleles used Sanger sequencing and the results showed perfect concordance (**Supplementary Figure S5**, **Supplementary Tables S2** and **S3**).

We then compared gAIRR-call results using PBMCs and mucosal cells from one individual to evaluate the impact of somatic recombination on the accuracy of gAIRR-call profiling. Out of 174 TRV alleles, 86 TRJ alleles and 22 IGJ alleles, the calling results from PBMCs and mucosal cells were identical for both known and novel alleles except one TRV allele (TRDV2*01), which was just above the adaptive threshold using mucosa data. In 396 known IGV alleles, there were only four disagreement between PBMCs and mucosal cells. Thus, we conclude that the combined gAIRR-seq and gAIRR-call pipeline is accurate in both known and novel alleles.

Further, we used gAIRR-call to analyze TRV alleles’ flanking sequences outside the allelic regions, which are important for the V(D)J recombination mechanism but have not been comprehensively collected in the database. Similar to verifying alleles, we utilized gAIRR-annotate to provide truth sets from whole-genome assemblies. Among the 224 alleles called with flanking sequences from HG001, there were 7 (3%) false positives and 0 false negatives; among the 222 alleles called from HG002, there were only 2 (1%) false positives and 0 false negatives. We further checked if the generated flanking sequences carried appropriate RSS, which were critical in V(D)J recombination. All 7 GIAB RMs and the primary cell sample carried identical or nearly identical RSS with known patterns in IMGT.

It is worth mentioning that we considered three gAIRR-seqed/gAIRR-called extended alleles correct though they did not align perfectly to the personal assemblies HG001 and HG002. We found that, after manual inspection, in all three instances the personal assemblies contained indel errors because of the PacBio sequencing weakness in homopolymers (**Supplementary 757** Section 8).

### 3.3 gAIRR-annotate could profile gAIRR alleles using genome assemblies

We collected 106 haplotypes from 6 works of literature (30–35) and annotated TR alleles using gAIRR-annotate. The dataset includes 36 individuals with a broad population background diversity. We first selected 12 high-quality samples to form a “HQ-12” set, where each sample could either be validated with family information or was curated with additional effort (Section 2.4). We showed that all haploids had 139-158 TRV alleles, and all but one haploid from HG002 had 5 TRD alleles and 82-86 TRJ alleles (**Supplementary Table S21**). gAIRR-annotate demonstrated HG002 to have fewer TR alleles, which is consistent with the known two long deletions in the paternal haplotype in the TRD and TRJ region (36) (Section 3.5). We then gAIRR-annotated the remaining 24 samples from HGSVC (31) and showed that the allele numbers were similar (**Supplementary Table S23**). We performed trio analysis for three annotated families (HG00514 with Southern Han Chinese ancestry; HG00733 with Puerto Rican ancestry; NA19240 with Yoruban ancestry). There were no Mendelian violations in TR loci for either known or novel alleles.

**Figure 4:**
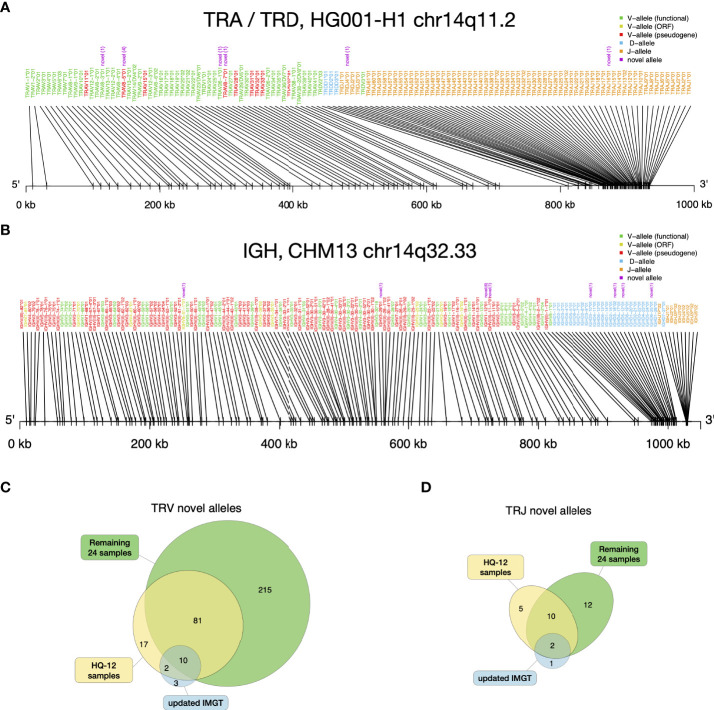
gAIRR-annotate results. **(A)** Positions of HG001’s TRA and TRD alleles determined by gAIRR-annotate. The purple text indicates that the allele is novel with its edit-distance compared to the most similar base allele inside parentheses. The text color of the gene names are basically according to IMGT’s color menu for genes (5). **(B)** Positions of CHM13 (36) IGH alleles determined by gAIRR-annotated. The figure settings are the same as in **(A)**. **(C)** TRV and **(D)** TRJ novel alleles found in HQ-12 samples (shown in yellow) and remaining 24 samples (shown in green) compared to the novel alleles updated in IMGT from v3.1.22 to v3.1.33.

The novel alleles discovered by gAIRR-annotate showed concordance with the gene locations of existing IMGT annotations. For example, we found a novel allele between TRAJ15*01 and TRAJ17*01 in HG001-H1. The novel allele had only one base difference compared to TRAJ16*01, which resides in the middle of *TRAJ15* and *TRAJ17* according to IMGT. Thus, we were confident that the allele was a novel allele of a known gene. On average, there were 18 novel V alleles and 3 novel J alleles in each haplotype (**Supplementary Table S21**). We examined the alleles’ relative positions for HG001 (**Figure 4A**) and HG002, and the TRA and TRD alleles’ relative positions were in the same pattern as the *Locus representation of IMGT (* 37) (**Supplementary Figure S7**). It shows that the novel alleles found by gAIRR-annotate, marked by purple text, are known genes with a limited number of genetic alterations, mostly single nucleotide substitutions, to the known allele cataloged in IMGT. We also verified the structural difference found by gAIRR-annotate by comparing it with IMGT. For example, both known structurally different combinations of TRGV alleles (12 and 14 genes) in IMGT were found in HG001’s haplotypes. The variations were in concordance with the IMGT gene locus map (**Supplementary Figure S8**).

Annotating IG genes is more challenging than TR genes because of somatic V(D)J recombination in EBV-transformed B lymphocytes. We first annotated the T2T-CHM13 genome (35) since it was not sequenced from B lymphocytes. We identified 32 novel alleles in the IGH, IGK, and IGL loci and 32 novel alleles in the orphons (**Figure 4B**; **Supplementary Table S8**). Similar to the analysis using gAIRR-call, we performed trio-analysis to validate the IG annotations. We classified an allele in the child genome as a “paternal”/“maternal” allele if an identical allele was observed only in the genome of their father/mother. If we observed the allele in both parents, we classified it as “identical”. We classified an V allele which overlapped with a V(D)J junction to be “post-V(D)J” **Supplementary Figure S2B**). We classified an allele to be a Mendelian violation (“violation”) if it had no direct evidence of V(D)J recombination and were not observed in the parent genomes. The Mendelian violation rates were 3.5% for known IGV genes (**Supplementary Table S5**), but novel allele calls had a lower quality (**Figure 3B**). The violations can result from mis-assemblies due to structural variations in IG regions and somatic V(D)J recombination.

We manually inspected the functional IGV annotations of HG001 to remove annotations in genes likely somatically V(D)J-recombined using evidence including the presence of V-J junction, abnormal structural variations, and short contig lengths. We then compared the annotations of functional IGHV genes with a dataset generated using capture-based long-read sequencing (19), and all but the *IGHV2-26* gene showed concordance. Sanger sequencing validation supported the allele called by gAIRR-annotated in *IGHV2-26* (**Supplementary Figure S5**). We used the curated HG001 IG annotations as the true allele types to assess the accuracy of gAIRR-call (Section 3.2).

### 3.4 Identified novel alleles are consistent with, and far outnumbering, the latest IMGT update

We identified a large number of novel alleles from the 36 samples, as compared with the IMGT database. We used IMGT v3.1.22 (April, 2019) as the baseline in this study, since it was the database we used to design gAIRR-seq probes. The latest version of the IMGT database (v3.1.33, March, 2021) had 15 TRV and 3 TRJ alleles added for human TR since v3.1.22. The novel alleles identified by gAIRR-annotate far outnumbered the IMGT updates (325 vs. 15 for TRV and 29 vs. 3 for TRJ; **Figures 4C, D**). Out of 325 novel TRV alleles, 167 are functional, 49 are open-reading frame, and 109 are pseudogenes. For the 29 novel TRJ alleles, 27 are functional and 2 are open-reading frame. For the alleles updated by IMGT, a large fraction of TRV and TRJ alleles (14 out of 18) was also discovered by gAIRR-annotate (**Figures 4C, D**), demonstrating high efficiency and accuracy of gAIRR Suite.

The gAIRR-annotate method can also annotate extended TR alleles, which we defined as the alleles plus 200-bp flanking sequences from both ends. We annotated 511 extended TRV alleles in the HQ-12 set, all of which were phased, and only 413 were known in the latest IMGT database. We further analyzed the RSS in the extended regions given their important participation in the V(D)J recombination mechanism. According to IMGT gene-DB, most TR RSSs follow the rule that the heptamer and nonamer in the RSS are separated by either a 23-bp (191 V alleles) or a 12-bp spacer (89 J alleles). However, there are some functional TR alleles having 24-bp (10 V alleles) or 22-bp (4 V alleles) spacers and occasionally 11-bp (TRAG20*01), 10-bp (TRAJ7*01) or 13-bp (TRDJ2*01) spacers. We used gAIRR-annotate to identify known RSS patterns in the 36 genomes and discovered 56 novel TRV and 20 novel TRJ RSSs (**Table 1**). We observed RSS polymorphism across different populations. For example, all 36 samples carry the IMGT TRAJ12*01 RSS and 6 out of 36 samples also carry a novel TRAJ12*01 RSS with a SNP at the nonamer (heterozygous).

**Table 1:** Number of known and novel TR RSS in the gAIRR-called and gAIRR-annotated flanking sequences.

	TRV				TRJ			
	#known		#novel		#known		#novel	
sample	Func	P/O	Func	P/O	Func	P/O	Func	P/O
HG001	94	43	4	1	66	10	7	1
HG002	95	43	6	1	65	10	9	1
HG003	94	44	3	1	66	10	6	1
HG004	91	43	7	1	65	10	8	1
HG005	93	44	8	2	67	10	7	1
HG006	96	46	6	1	67	10	8	1
HG007	95	44	7	2	67	10	5	1
Primary cell sample	95	43	6	2	67	10	6	1
HQ-12 set samples	96	46	14	3	67	10	11	3
HGSVC-additional-24 samples	96	46	31	22*	67	10	15	3

*: In addition to 22 pseudogene and ORF alleles with novel RSS, we didn’t find any appropriate nonamers at TRBV24/OR9-2*03 (ORF) for HG01505. The number of RSS known in IMGT (5) and novel RSS are shown in columns #known and #novel respectively. The functionality Func indicates that the RSSs come from functional genes while P/O indicates that the RSSs come from pseudogenes or open reading frames (ORFs). The RSS from HG001-7 and the primary cell sample are called from both gAIRR-seq and gAIRR-call while the RSS from HQ-12 and the additional 24 samples are called from gAIRR-annotate alone.

We built a novel-allele database collecting all identified novel alleles, an extended-allele database collecting alleles with 200-bp flanking sequences, and an RSS database collecting RSS information in flanking sequences for both TRV and TRJ (see **Supplementary Table 1**). We showed that we could broadly expand the size of known TR databases using gAIRR-annotate and whole-genome assemblies. With the rapidly increasing number of assemblies utilizing accurate long-read sequencing, we are optimistic that gAIRR-annotate will help the community uncover TR germline DNA information at a much greater speed than traditional methods.

### 3.5 Two structural variants in HG002 identified by gAIRR-annotate and validated with gAIRR-seq

**Figure 5:**
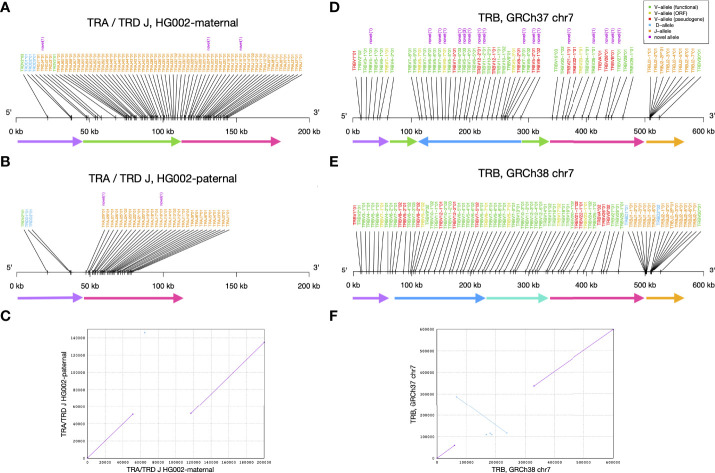
Structural variants identified by gAIRR-annotate. **(A–C)** The 65 kbp structural variation in TRA and TRD J region of HG002. **(A)** The maternal haplotype, **(B)** the paternal haplotype, and **(C)** the sequence alignment of HG002’s maternal and paternal TRA/TRD J sequence. In **(A, B)** the arrows with the same color can be aligned between the two haplotypes. The green arrow is the segment deleted in paternal haplotype. **(D–F)** The inversion and deletion of TR beta chain germline genes of the reference genome. **(D)** GRCh37 chr7, **(E)** GRCh38 chr7, and **(F)** the sequence alignment of GRCh37 and GRCh38 at TR beta chain. In **(D, E)**, the arrows with the same color can be aligned between the two haplotypes. The deep blue arrow indicates the inversion between the reference genomes.

**Figure 6:**
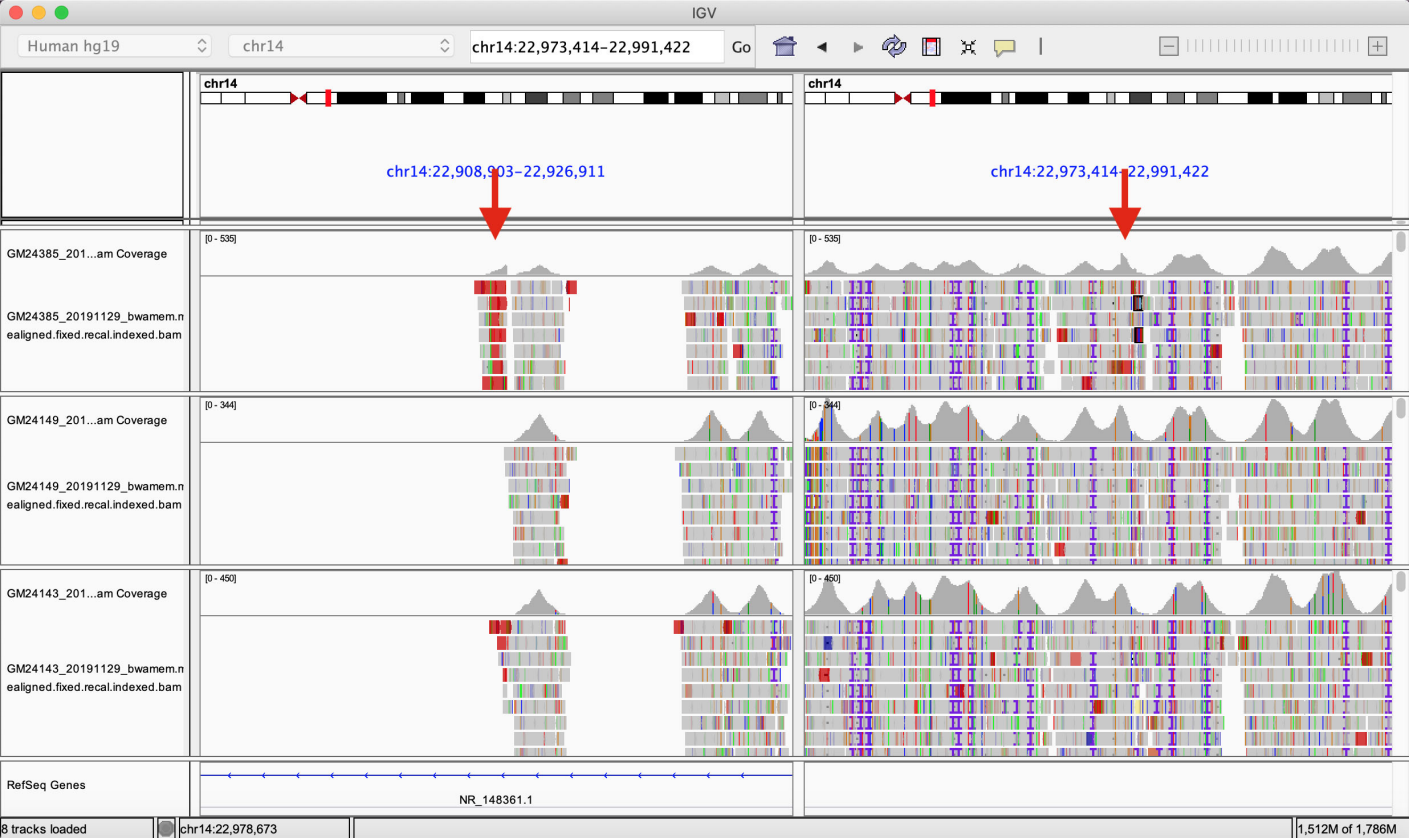
The Integrated Genomics Viewer visualization of HG002’s structural variant. The Integrated Genomics Viewer visualization of the capture-based reads from HG002 (son, top), HG003 (father, middle), and HG004 (mother, bottom) aligned to GRCh37 chromosome 14. There are two red arrows indicating abrupt read-depth changes of HG002’s reads at chr14:22,918,113 and chr14:22,982,924.

When visualizing alleles identified by gAIRR-annotate, we observed a 65 kbp deletion in TRA/TRD at chr14:22,918,114-22,982,924 using GRCh37 or chr14:22,449,122-22,513,942 using GRCh38. The deletion contained 1 D, 1 V, and 36 J genes from *TRDD3* to*TRAJ30* in the paternal haplotype (**Figures 5A–C**). We noticed low numbers of TRJ and TRD alleles in the paternal haplotype of HG002 (**Supplementary Table S21**). To further verify the 65 kbp deletion, we performed trio analysis using gAIRR-seqed reads from the HG002-HG003-HG004 family (**Figure 6**). We noticed reads split across the breakpoints and reduced alignment coverage in the deleted region unique to HG002, suggesting a *de novo* deletion (Section 2.6). The deletion contributed to a loss of 32 TRAJ genes and all 4 TRDJ genes from the paternal haplotype. The GIAB structure variant report for HG002 (36) also showed a deletion at the same locus, where a 13 bp fragment in HG002 replaces a 64,807 bp fragment in GRCh37.

Similarly, we noticed a 10 kbp deletion in the paternal haplotype of HG002 at chr7:142,494,032-142,494,532 using GRCh37 or chr7:142,786,222-142,796,848 using GRCh38. Note that GRCh37 has 10,125 bps deleted with respect to GRCh38 at this locus. This deletion contained 2 D and 9 J alleles from *TRBD1* to *TRBJ2-2P* (**Supplementary Figure S9B**). We verified the deletion with gAIRR-annotate, gAIRR-seq, and the report from GIAB (36) (**Supplementary Section 13**). The GIAB report showed 0/0 genotypes for HG003 and HG004 at both the chr14 and chr7 deletions, indicating *de novo* events.

### 3.6 gAIRR-annotate uncovered substantial differences in GRCh37 and GRCh38

We identified an inversion in GRCh37 with respect to the GRCh38 primary assembly that covered TRBV alleles (**Figures 5D–F**). In this inversion, there were 39 TRBV genes (*TRBV6-2* to *TRBV18* following the gene order provided by IMGT) successfully annotated in GRCh38. In contrast, in GRCh37, only 27 genes from *TRBV8-1* to *TRBV7-8* were successfully annotated, but with a reversed order. We observed another long missing sequence (about 10 kbp) in GRCh37 in the TRB region, covering about half of the expected TRBD and TRBJ genes. We noticed GRCh38 didn’t carry this deletion, and we annotated reasonable numbers of TRBD and TRBJ genes. When comparing annotated alleles to the IMGT database, we showed that on chromosome 7, all alleles in GRCh38 are known, and there are 18 novel alleles in GRCh37. In IG alleles, there are also some differences between the reference genomes; however, all the genome structures are in consistence with IMGT’s locus representation. (**Supplementary Figure S12**; **Supplementary Table S26**)

We further analyzed an GRCh38 alternative contig, *chr7_KI270803v1_alt*, which contained TRBV alleles (**Supplementary Figure S13**). We identified eleven novel TRBV alleles in *chr7_KI270803v1_alt* using gAIRR-annotate. Compared to the GRCh38 primary assembly, *chr7_KI270803v1_alt* has six additional TRBV genes. TRB genes in both chr7 and *chr7_KI270803v1_alt* showed concordance with the *locus representation* of IMGT (37). Thus, when a personal assembly is not available, we suggest using GRCh38 instead of GRCh37 for TR analysis in that GRCh38 shows concordance with the IMGT database in sequence completeness and carries better-known alleles.

## 4 Discussion

We have developed the gAIRR Suite that includes probe capture-based targeted sequencing and computational analyses to profile germline TR and IG genes. gAIRR-seq can enrich the TR and IG regions with high on-target rates and sufficient read depth, and gAIRR-call can then accurately call both known and novel alleles using gAIRR-seq reads. gAIRR-annotate can take advantage of whole genome assemblies to accurately annotate TR and IG genes in both allelic and flanking regions. We applied gAIRR-annotate to identify 325 novel alleles in TRV (167 are functional) and 29 novel alleles in TRJ (27 are functional) from 36 subjects. We identified RSSs with spacers neither 12-bp nor 23-bp in length, which had been reported before (38, 39) but not yet comprehensively profiled. gAIRR-annotate can also help to discover structural variants. We independently identified and then verified two known structural variants in the TR regions in HG002. We also searched for structural variants in the IG regions of CHM13, and found that the genome structures are consistent with IMGT’s locus presentation. Similarly, we used gAIRR-annotate to uncover substantial differences between references GRCh37 and GRCh38.

gAIRR-seq is advantageous in its high resolution and comprehensiveness. Compared to multiplex PCR-based methods, gAIRR-seq covers all known TR and IG genes and alleles in one experiment. Furthermore, novel alleles can also be identified because the probes can tolerate sequence mismatch to a certain degree. Each sample only costs approximately USD 200 for library preparation, target enrichment, and sequencing while all IG and TR genes/alleles being gAIRR-seqed in one experiment using easily available genomic DNA from cell lines or primary cells, such as PBMCs or mucosal cells. We envision that gAIRR-seq capture probe design can be further upgraded by gAIRR-annotate results thanks to the collection of accurate extended alleles. In this study, our probe design was limited to only V and J genes due to insufficient allele lengths for other genes. With a comprehensive population genomic TR and IG database becoming available, we plan to include TRD and IGD genes in future gAIRR-seq probe design.

Profiling germline IG genes is challenging because these regions could be somatically V(D)J-recombined in EBV-transformed B lymphocytes (19, 40). We manually curated the IG annotations of HG001 to evaluate the gAIRR-seq-call pipeline. Thanks to the high-coverage reads from gAIRR-seq, our pipeline was accurate in functional IGV genes except for those with substantial somatic loss of alleles. The gAIRR-seq-call pipeline is the first short-read-based approach capable of profiling all functional germline IGV genes to the best of our knowledge. The somatic variations in the cell lines reduced the accuracy of gAIRR-annotate. In the future, it is important to improve assembly algorithms or design post-assembly filtration methods to remove contigs that are mis-assembled or somatically V(D)J-recombined.

We also explored an alternative approach that using primary cells for IG experiments. We gAIRR-seqed the genomic DNA from both PBMCs and mucosal cells of a Taiwanese subject (**Supplementary Section 17**) and gAIRR-called similar numbers of known and novel alleles in the TRV and TRJ regions compared to the GIAB RMs. We reason that, in PBMCs, B lymphocytes and monocytes can provide un-recombined V(D)J genes for TR sequencing, and T lymphocytes and monocytes can provide un-recombiend V(D)J genes for IG sequencing. Since the gAIRR-call result on the PBMCs and mucosal cells show high concordance, utilizing primary cells (either PBMC or mucosal cells) for IG and TR genotyping is valid.

Up to now, it is almost insurmountable to study the impact of germline variations in TR and IG genes on human immune-related phenotypes and diseases. The single nucleotide polymorphism (SNP) genotyping array widely used for genome-wide association study (GWAS) tags only a tiny fraction of gAIRR variations (3, 13, 15). Whole-genome sequencing (WGS) can theoretically cover gAIRR variations; however, with neither a powerful bioinformatics tool (such as gAIRR-call) nor reliable reference genome(s) (41), WGS has not yet been successfully used to retrieve gAIRR information for human genetic study due to high genomic complexity in the gAIRR regions and relatively lower sequencing depth compared to targeted sequencing. Whether germline TR and IG variations have a broad and strong impact on human immune-related phenotypes and diseases, just like the human leukocyte antigen (HLA) variations do (42, 43), is an attractive question awaiting more studies. In our view, today’s status of germline TR and IG genes investigations are similar to the situation of SNPs before the International HapMap Project (44), or HLA before Sanger sequencing-based typing (SBT). We badly need a HapMap equivalent for germline TR and IG genes.

We envision that gAIRR Suite can help in at least the following scenarios. First, the discovered novel alleles can facilitate development of better SNP arrays and better WGS analysis references of gAIRR in the future. Second, the personal germline TR and IG genes profile can be directly employed for genetic study and clinical applications. Third, the personal germline TR and IG genes, together with other critical immune genes (such as HLA and killer cell immunoglobulin-like receptor (KIR) genes), can be tested for di-genic or oligo-genic effects on various immune-related phenotypes. Last but not least, the combined analysis of germline TR and IG genes and dynamic mRNA-based AIRR profiles might help decipher molecular mechanisms underlying immunological responses.

## Data availability statement

The gAIRR-seq sequence data of the seven reference materials and two primary cell samples have been deposit in SequenceRead Archive https://www.ncbi.nlm.nih.gov/sra/PRJNA767687. The source code of gAIRR Suite is publicly available on GitHub at https://github.com/maojanlin/gAIRRsuite under the MIT license. The designed gAIRR-seq probes, annotated novel alleles, flanking sequences, and RSS databases are available in the **Supplementary Table 1**. The annotation bed files of human reference genome GRCh37, GRCh38, and CHM13 are available at https://github.com/maojanlin/gAIRRsuite/tree/master/supplementary_files/reference_genome_annotation (Details in Table 1.XLXS).

## Ethics statement

The studies involving human participants were reviewed and approved by Research Ethics Committee of the National Taiwan University Hospital (201611020RIND). The patients/participants provided their written informed consent to participate in this study.

## Author contributions

Y-CL and P-LC designed the sequencing experiments and AL performed the experiments. Y-CL and N-CC designed the preliminary analysis pipeline. M-JL designed the final analysis pipelines and wrote the software. M-JL, N-CC, and Y-CL interpreted all the analysis results with P-LC’s guidance. SK, JH, C-YC, C-LH, W-SY, and P-LC initiated this project and assisted with discussions. M-JL, N-CC, and P-LC wrote the manuscript with input from all authors. All authors read and approved the final manuscript.

## Funding

This study was supported by Taiwan Ministry of Science and Technology grants (MOST 108-2314-B-002-069-MY3) and National Taiwan University Hospital (111-UN0061, 109-S4521, 108-T05).

## Acknowledgments

We thank to National Core Facility for Biopharmaceuticals (NCFB, MOST 108-2319-B-492 -001) for support and National Center for High-performance Computing (NCHC) of National Applied Research Laboratories (NARLabs) in Taiwan for providing computational and storage resources. We thank the staff of the Second Core Lab, Department of Medical Research, National Taiwan University Hospital for technical support during the study. We also thank Justin Zook, Yana Safonova, Michael Ford, and Ananth Hari for their helpful suggestions in sequencing experiments and analysis.

## Conflict of interest

The authors declare that the research was conducted in the absence of any commercial or financial relationships that could be construed as a potential conflict of interest.

## Publisher’s note

All claims expressed in this article are solely those of the authors and do not necessarily represent those of their affiliated organizations, or those of the publisher, the editors and the reviewers. Any product that may be evaluated in this article, or claim that may be made by its manufacturer, is not guaranteed or endorsed by the publisher.
