# Supplementary material for: Profiling genes encoding the adaptive immune receptor repertoire with gAIRR Suite
Source: Front Immunol. 2022 Sep 8;13:922513. doi: 10.3389/fimmu.2022.922513 (PMC9496171; doi:10.3389/fimmu.2022.922513)
Supplement: Supplementary file 1 [file DataSheet_1.pdf]

Supplemental Materials

1 gAIRR-seq's result on 7 RMs and two primary cell samples

Table S1. The number of reads sequenced, fraction of aligned reads and on-target rate of gAIRR-seq using samples HG001 and HG002. The target region is defined as the union of annotated allelic regions, where each region includes the allele and 800-bp flanking sequences extended from both sides as shown in Supplementary Fig. S17. RM HG003-7 and the primary cell sample are not applicable in capture-efficiency analysis for lacking personal assembly annotation.

Sample	# of reads sequenced	% of reads aligned (H1/H2)	% in target region (H1/H2)
HG001	280,579	97.6/98.0	83.3/83.7
HG002	281,494	98.5/96.1	84.5/83.3
HG003	307,312	N/A	N/A
HG004	362,271	N/A	N/A
HG005	327,354	N/A	N/A
HG006	338,361	N/A	N/A
HG007	297,186	N/A	N/A
Primary cell sample	283,691	N/A	N/A

We located all TRV, TRJ, and IGV alleles in the assemblies with gAIRR-annotate to validate if the reads are in AIRR regions. Considering the fragment lengths are up to 800 bp in our sequencing method, we defined a read aligned within 800 bp from its allele boundaries to be on-target.

We assessed the biases of probe preferences during library preparation using probe capture-based enrichment. The only bias we observed is an extremely long TRV pseudogene *TRAV8-5*. The gene length is 1355 bp, while the second-longest TRV gene is only 352 bp. The read-depth coverage of *TRAV8-5*, 270×, is relatively low compared to other V alleles, 405× on average. gAIRR-call tackles the uneven problem by making *TRAV8-5* a special case in adaptive threshold generation (Section 2.2). The lower read coverage of *TRAV8-5* is likely due to the high allele length-to-probe ratio. By adding more probes according to the allele length, the bias can probably be solved.

2 The novel TRV and TRJ allele relationship in the Ashkenazi trio and Chinese trio

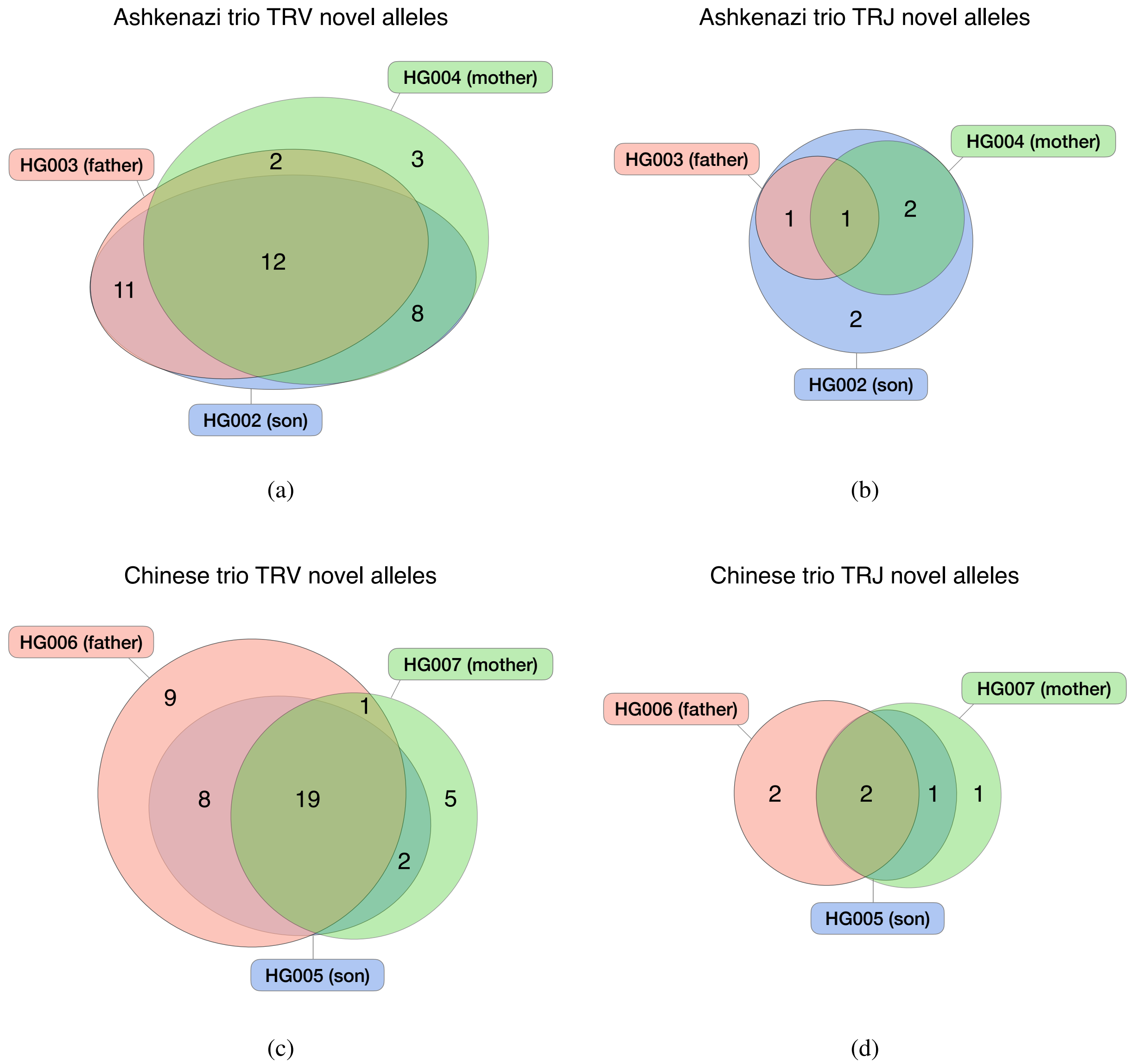

Fig. S1. Mendelian relationship of novel alleles. a, TRV and **b,** TRJ regions in the Ashkenazi family. HG002: son; HG003: father; HG004: mother. In the TRV region, there are no unique alleles owned by HG002. In TRJ, HG002 owns two unique TRJ alleles due to *de novo* structural variants (Section 3.5). The **c,** TRV and **d,** TRJ regions in the Chinese family. HG005: son; HG006: father; HG007: mother.

3 gAIRR-seq shows read depth drop at IGLV loci of HG001

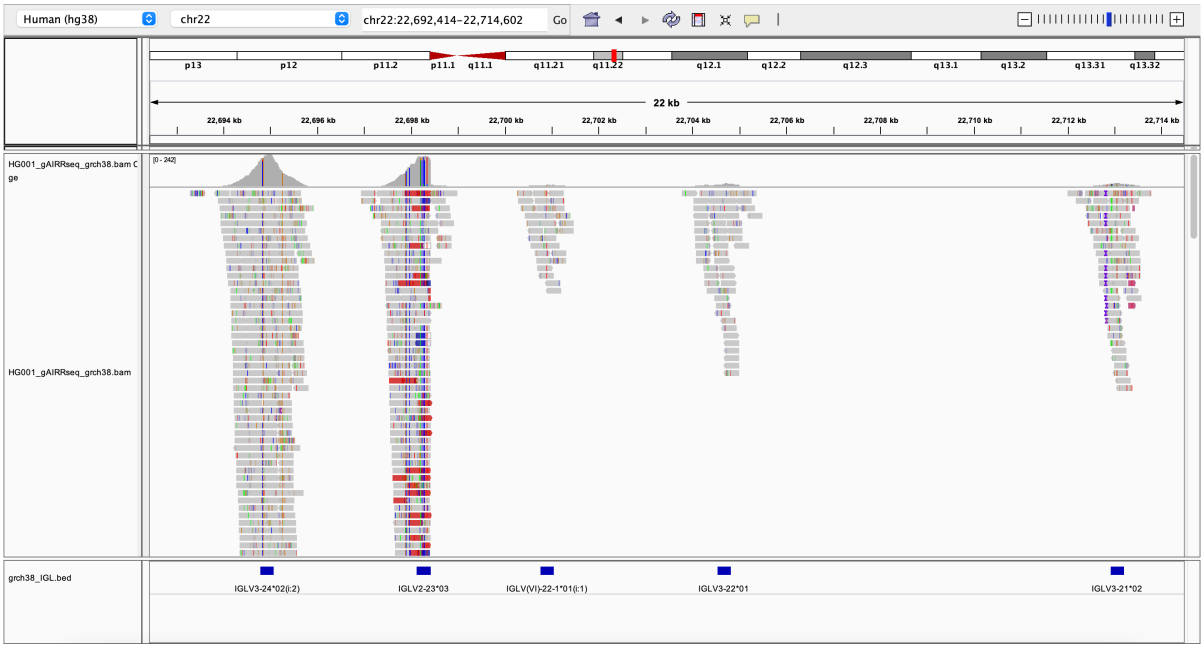

(a)

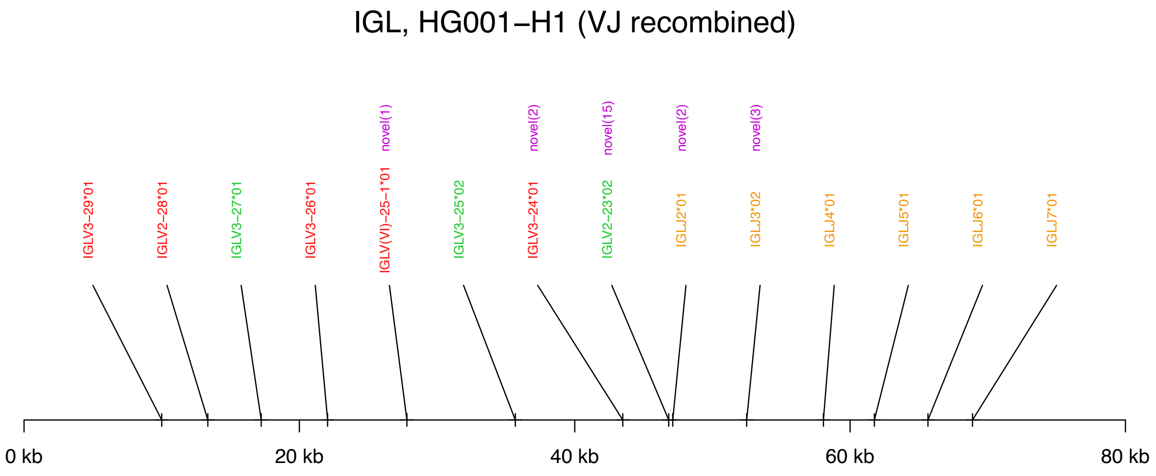

(b)

Fig. S2. a, the Integrated Genomics Viewer[45] visualization of the HG001 position where gAIRR-seq shows read depth drop at the IGLV locus. The reference is GRCh38. The read depth drop on the 5' end after IGLV2-23 was probably due to VJ recombination at IGLV2-23 in the cell line. **b,** the gAIRR-annotate result on the same position in one of the contig from HG001's assembly[31]. The contig also show a VJ recombination between IGLV2-23 and IGLJ2.

4 gAIRR-annotate shows novel IGV alleles result from post-V(D)J recombination of HG002

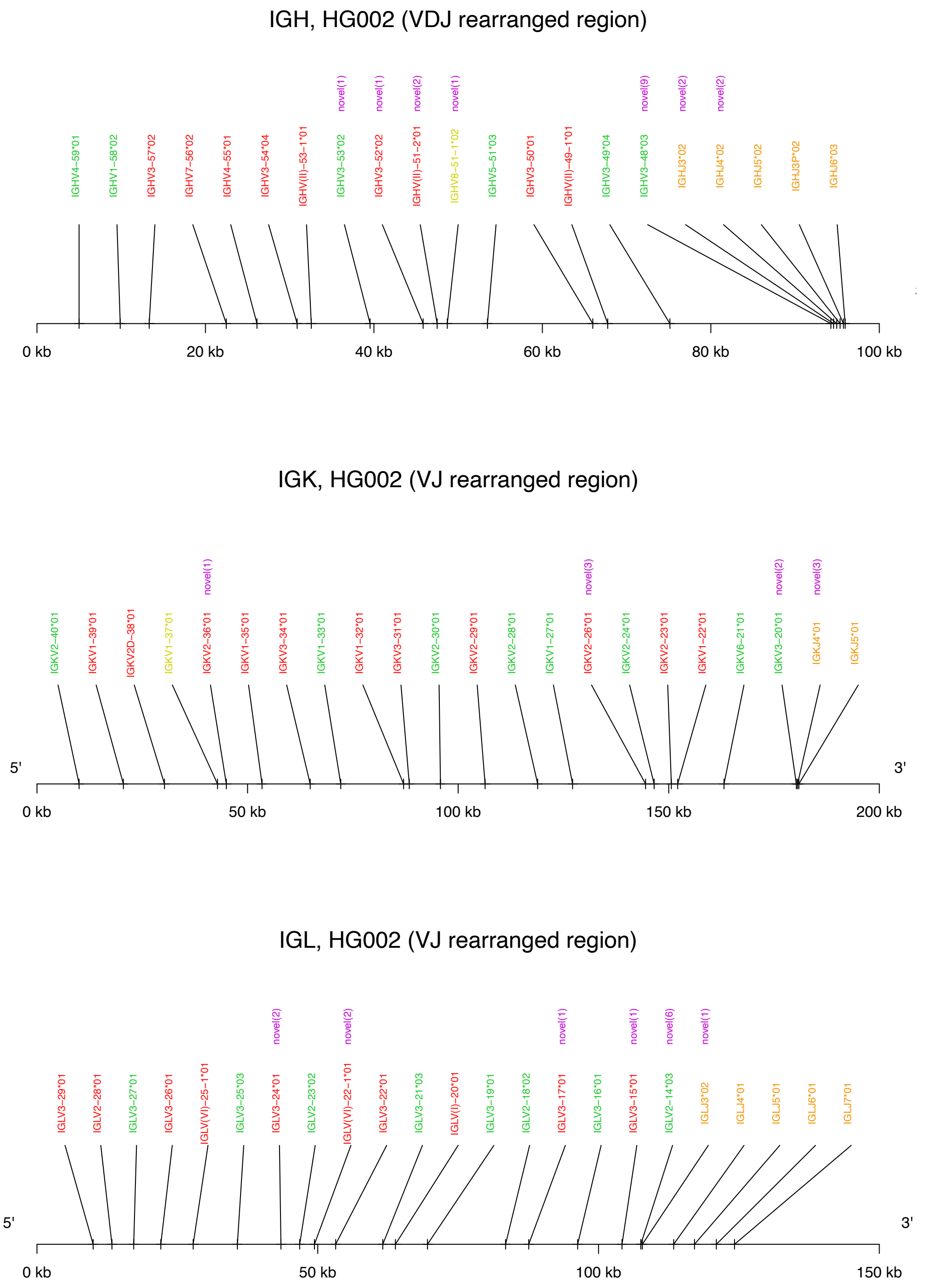

Fig. S3. From top to bottom are the gAIRR-annotated contigs showing that the alleles of HG002's 4 genes *IGHV3-48*, *IGKV3-20*, and *IGLV2-14* being post-V(D)J product.

5 Mendelian violation due to read coverage drop in IGHV trio analysis

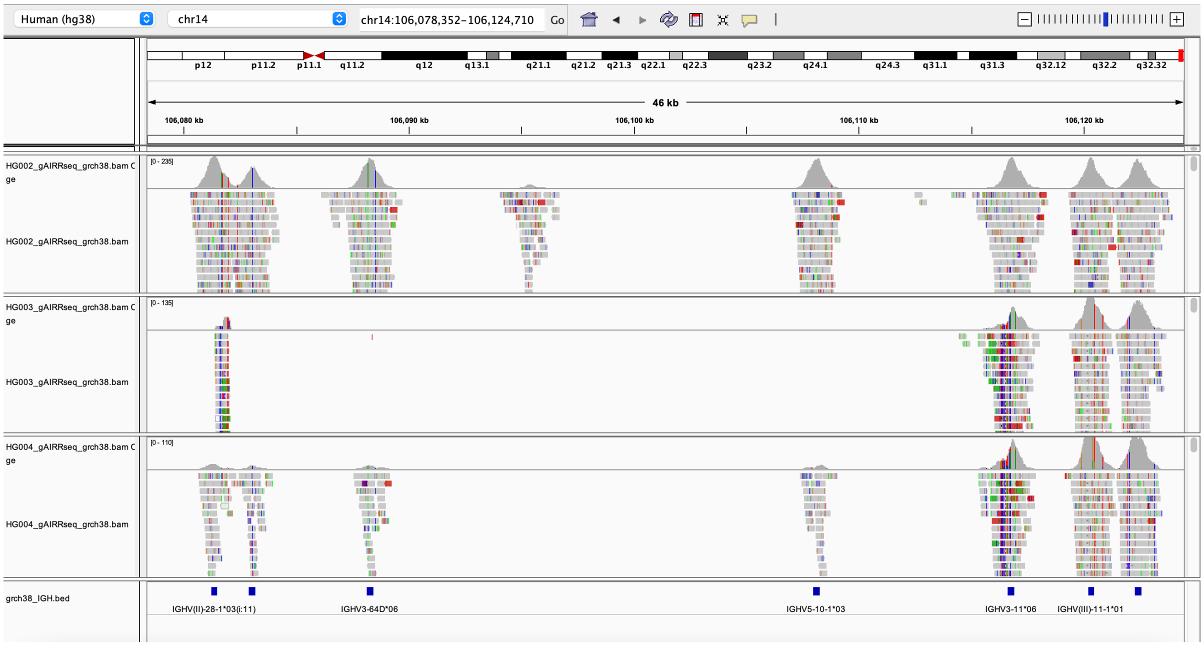

Fig. S4. A Mendelian violation in gAIRR-seq-call analysis. We visualized the alignments against GRCh38 for an Ashkenazim Jewish family using Integrated Genomics Viewer[45]. Top: HG002 (son); middle: HG003 (father); bottom: HG004 (mother). Functional genes *IGHV3-64D* and *IGHV5-10-1* were observed in HG002 and HG004, but gAIRR-call did not call these genes in HG004 due to read coverage drops. The sequenced HG004 data had low coverage in both genes, suggesting many cells had been VDJ rearranged.

6 Sanger Sequencing Validation on novel alleles

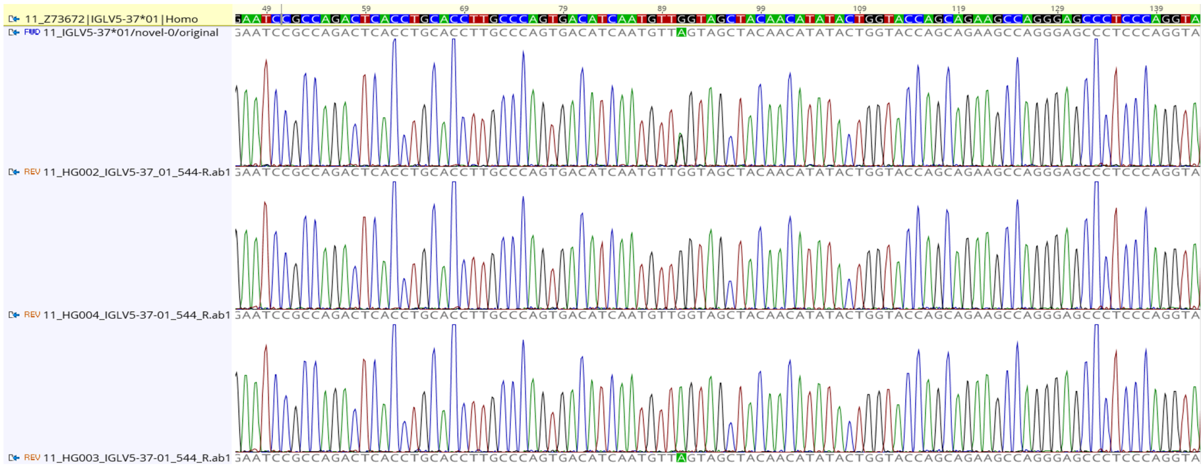

(a)

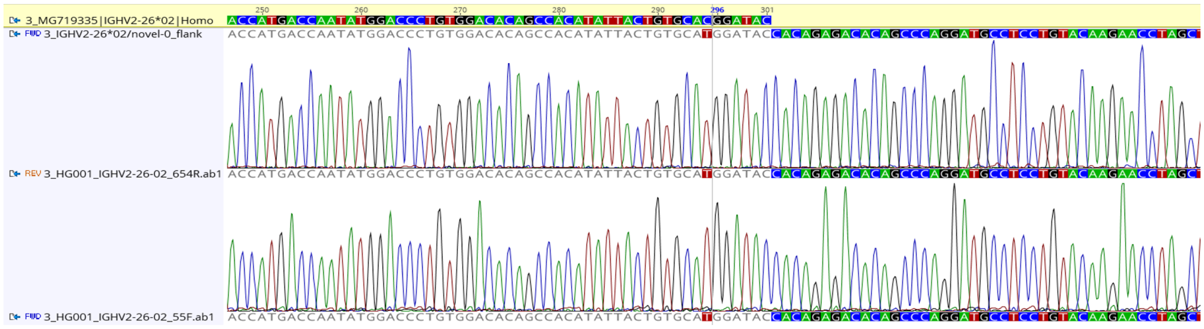

(b)

Fig. S5. **a,** an example of Sanger sequencing validation on IGLV5-37 novel genes in HG002 family. The novel allele G>A at position 91 only exist in HG002 and HG003, which is in concordance with gAIRR-call result. **b,** the Sanger sequencing validation on HG001's novel IGHV2-26 gene. The novel allele has a C>T on position 295 different from IGHV2-26*02.

Table S2. The 4 novel IGLV alleles and 8 novel TRAV alleles in HG002 family validated with Sanger sequencing. The number in the parenthesis of after the allele name indicates how many nucleotide the novel allele is different from the known allele.

	HG002	HG003	HG004
IGLV1-41*01(2)	1	1	0
IGLV5-37*01(1)	1	1	0
IGLV6-57*01(2)	1	1	1
IGLV7-46*01(1)	1	1	1
TRAV1-2*01(1)	1	1	0
TRAV25*01(1)	1	1	1
TRAV27*01(1)	1	1	0
TRAV30*01-0(1)	1	0	1
TRAV30*01-1(2)	1	1	0
TRAV35*01(2)	1	0	1
TRAV8-1*01(1)	1	1	0
TRAV8-3*02(1)	1	1	1

Table S3. The 5 novel IGLV alleles and 4 novel TRAV alleles in HG005 family validated with Sanger sequencing. The number in the parenthesis of after the allele name indicates how many nucleotide is different between the novel allele and the known allele.

	HG005	HG006	HG007
IGLV3-16*(1)	1	1	0
IGLV3-21*01(1)	1	1	1
IGLV3-32*01(1)	1	0	1
IGLV6-57*01(1)	1	1	0
IGLV7-46*01(1)	1	0	1
TRAV26-2*01(1)	1	1	0
TRAV28*01(1)	1	1	1
TRAV6*02(1)	1	1	0
TRAV8-3*02(1)	1	1	1

Table S4. The 2 IGLV alleles and 5 novel TRAV alleles in HG001 validated with Sanger sequencing. The number in the parenthesis of after the allele name indicates how many nucleotide is different between the novel allele and the known allele.

HG001
IGHV2-26*02(1)
IGHV1-46*04
TRAV8-3*02(1)
TRAV23/DV6*02(1)
TRAV30*01(1)
TRAV35*02(2)
TRAV36/DV7*01(1)

7 Trio Validation Details

Table S5. Trio validation details. The genes with V(D)J recombination evidence and genes without parents' information (due to gene lost) are classified as Limited Evidence.

Novelty	Chain	Sample	Violation	Paternal	Maternal	Identical	Limited Evidence
Known	TRV	HG002	0	19	26	112	0
Known	TRV	HG005	0	3	7	127	0
Known	TRV	HG00733	0	18	17	128	0
Known	TRV	NA19240	0	14	11	127	0
Known	TRV	HG00514	0	12	11	126	0
Known	TRJ	HG002	0	0	2	81	0
Known	TRJ	HG005	0	0	0	83	0
Known	TRJ	HG00733	0	2	3	83	0
Known	TRJ	NA19240	0	2	1	83	0
Known	TRJ	HG00514	0	4	3	80	0
Known	IGV	HG002	3	17	5	94	1
Known	IGV	HG005	0	6	11	113	0
Known	IGV	HG00733	3	20	13	82	66
Known	IGV	NA19240	7	30	5	62	33
Known	IGV	HG00514	2	10	2	104	20
Novel	TRV	HG002	0	11	8	12	0
Novel	TRV	HG005	0	8	2	19	0
Novel	TRV	HG00733	0	10	7	4	0
Novel	TRV	NA19240	0	12	15	15	0
Novel	TRV	HG00514	0	7	5	7	0
Novel	TRJ	HG002	2	1	2	1	0
Novel	TRJ	HG005	0	0	1	2	0
Novel	TRJ	HG00733	0	2	0	1	0
Novel	TRJ	NA19240	0	2	2	2	0
Novel	TRJ	HG00514	0	1	2	2	0
Novel	IGV	HG002	3	2	0	2	5
Novel	IGV	HG005	0	6	4	10	0
Novel	IGV	HG00733	0	6	0	1	10
Novel	IGV	NA19240	0	4	6	5	2
Novel	IGV	HG00514	3	3	6	3	3

Table S6. IGHV alleles in HG002 trio. The alleles violate the Mendelian rule is highlighted in bold. The alleles without enough parent information are highlighted in italic. "Post-V(D)J" indicates the allele with evidence of V(D)J rearrangement.

Gene	HG002	HG003	HG004
IGHV7-81	01	01	01
IGHV3-74	01	01	01
IGHV3-73	01/02	01/02	01/02
IGHV3-72	01	01	01
IGHV2-70	01/**Novel(0)**	Novel(1)/Novel(2)/Novel(3)	01/05/Novel(4)/Novel(5)
IGHV1-69D	lost	lost	lost
IGHV1-69-2	lost	lost	lost
IGHV2-70D	lost	lost	lost
IGHV1-69	01/04	01/04	01/02
IGHV3-66	01/03	01/03	02/03
IGHV3-64	01/02	01/02	01/02
IGHV4-61	01	01	01/02/Novel
IGHV4-59	01/08	01/08	01
IGHV1-58	02	01/02	01/02
IGHV3-53	01	01/02	01/02/04
IGHV5-51	03	01/03	01/03
IGHV3-49	04	04	03
IGHV3-48	Post-VDJ	03	01/04
IGHV1-46	lost	01	01/03
IGHV1-45	lost	02	02
IGHV3-43	lost	01	01
IGHV4-39	lost	01	01/07
IGHV1-38-4	lost	lost	01
IGHV3-38-3	lost	lost	01
IGHV3-43D	lost	04	03
IGHV4-38-2	lost	01	02
IGHV3-38	02	02	02
IGHV3-35	01	01	01/Novel
IGHV4-34	01	01	01/03
IGHV3-33	01	01	01/06
IGHV4-31	03	03	03
IGHV3-30-5	01	01	01
IGHV4-30-4	lost	01	lost
IGHV3-30-3	01	01	01/03
IGHV4-30-2	01	01/05	01/04
IGHV4-30-1	lost	lost	lost
IGHV3-30	18	18	04/18
IGHV4-28	01	01	01/Novel
IGHV2-26	01	01	01/Novel
IGHV1-24	01	01	01/Novel
IGHV3-23D	01	01	01
IGHV3-23	01	01	01/Novel
IGHV3-21	01	01	01
IGHV3-20	**02**	01/04	01
IGHV1-18	04	01/04	01
IGHV3-16	02	02	02
IGHV3-15	01	01/07	01
IGHV3-13	05	01	01/05
IGHV3-11	**06**	01	01

Continued on next page

Table S6 – *Continued from previous page*

IGHV3-9	lost	01	01
IGHV1-8	lost	01	01
IGHV5-10-1	*03*	lost	lost
IGHV3-64D	*Novel*	lost	lost
IGHV3-7	**Novel**	01	01
IGHV2-5	01	01/02	02
IGHV7-4-1	01	01	02
IGHV4-4	02	02	01/03
IGHV1-3	01	01	01
IGHV1-2	04	04	05
IGHV6-1	01	01	01

Table S7. IGKV alleles in HG002 trio. The alleles violate the Mendelian rule is highlighted in bold. The alleles without enough parent information are highlighted in italic. "Post-V(D)J" indicates the allele with evidence of V(D)J rearrangement.

Gene	HG002	HG003	HG004
IGKV3D-7	01	01	01
IGKV1D-8	01	01	01/02
IGKV1D-43	01	01	01
IGKV1D-42	01	01	01/02
IGKV3D-11	01	01	01/02
IGKV1D-12	01	01	01/02
IGKV1D-13	01	01	01/02
IGKV3D-15	01	01	01/03
IGKV1D-16	01	01	01
IGKV1D-17	01	01	01
IGKV6D-41	01	01	01
IGKV3D-20	01	01	01
IGKV6D-21	01/02	01/02	01/02
IGKV2D-24	01	01	lost
IGKV2D-26	03	03	lost
IGKV2D-28	01	01	01
IGKV2D-29	01	01	01
IGKV2D-30	01	01	01
IGKV1D-33	01	01	01
IGKV1D-37	01	01	01
IGKV1D-39	01	01	01
IGKV2D-40	01	01	01
IGKV2-40	01	01	01
IGKV1-39	01	01	01
IGKV1-37	01	01	01
IGKV1-33	01	01	01
IGKV2-30	01	01	01
IGKV2-29	01	01	01
IGKV2-28	01	01	01/Novel
IGKV1-27	01	01	01
IGKV2-24	01	01	01
IGKV6-21	01	01	01
IGKV3-20	Post-VJ	01	01
IGKV1-17	lost	01	01
IGKV1-16	lost	01/02	02
IGKV3-15	lost	01/Novel	01
IGKV1-13	lost	01	01/02
IGKV1-12	lost	01	01/02
IGKV3-11	lost	01	01
IGKV1-9	lost	01	01
IGKV1-8	lost	01	01
IGKV3-7	lost	04	04/04/novel-0
IGKV1-6	lost	01	01
IGKV1-5	lost	03	03
IGKV5-2	lost	01/Novel	01
IGKV4-1	lost	Novel(0)/Novel(1)	01

Table S8. IGLV alleles in HG002 trio. The alleles violate the Mendelian rule is highlighted in bold. The alleles without enough parent information are highlighted in italic. "Post-V(D)J" indicates the allele with evidence of V(D)J rearrangement.

Gene	HG002	HG003	HG004
IGLV4-69	01	01	01
IGLV8-61	01	01	01
IGLV4-60	03	03	03
IGLV6-57	02/Novel	02/Novel	02/Novel
IGLV11-55	02	01/02	02
IGLV10-54	01/02	01	01/02
IGLV5-52	01	01	01
IGLV1-51	01	01	01
IGLV1-50	01	01	01
IGLV9-49	01	01	01
IGLV5-48	01	01	01/02
IGLV1-47	01	01/Novel	01/02
IGLV7-46	Novel	01/Novel	01/Novel
IGLV5-45	02	02/Novel(0)	03/Novel(1)
IGLV1-44	01	01	01
IGLV7-43	01	01	01
IGLV1-41	01/Novel	01/Novel	02
IGLV1-40	01	01	01
IGLV5-39	01	01	01
IGLV5-37	01/Novel	Novel	01
IGLV1-36	01	01	01
IGLV2-33	01	01	01
IGLV3-32	01	01	01
IGLV3-27	01	01	01
IGLV3-25	03/**Novel**	03	03
IGLV2-23	02/03	03	02
IGLV3-22	01	01	01
IGLV3-21	02/**03**	02	Novel
IGLV3-19	01	01	01
IGLV2-18	02	01/02	02
IGLV3-16	01	01	01
IGLV2-14	04/Post-VJ	01/04	lost
IGLV3-12	01	01/02	lost
IGLV2-11	01	01	01
IGLV3-10	01	01	01
IGLV3-9	01	01	01
IGLV2-8	01	01	01
IGLV4-3	01	01	01
IGLV3-1	*Novel*	lost	lost

Table S9. IGHV alleles in HG005 trio. The alleles violate the Mendelian rule is highlighted in bold. The alleles without enough parent information are highlighted in italic. “Post-V(D)J” indicates the allele with evidence of V(D)J rearrangement.

Gene	HG005	HG006	HG007
GHV7-81	01	01	01
IGHV3-74	01	01	01/Novel
IGHV3-73	01	01	01
IGHV3-72	01	01	01
IGHV2-70	15	15	15/Novel
IGHV1-69D	lost	lost	lost
IGHV1-69-2	lost	lost	lost
IGHV2-70D	lost	lost	lost
IGHV1-69	04	04	04
IGHV3-66	01	01	01
IGHV3-64	01	01	01/Novel
IGHV4-61	lost	lost	lost
IGHV4-59	01/08	01/08	01/08
IGHV1-58	02	02	02
IGHV3-53	04	01/04	04
IGHV5-51	01	01	01
IGHV3-49	04	04/05	04
IGHV3-48	03	02/03	03
IGHV1-46	01	01	01
IGHV1-45	03	02/03	03
IGHV3-43	02	01/02	02
IGHV4-39	01	01	01
IGHV1-38-4	lost	lost	lost
IGHV3-38-3	lost	lost	lost
IGHV3-43D	lost	lost	lost
IGHV4-38-2	lost	lost	lost
IGHV3-38	02/03	02	02/03
IGHV3-35	01	01	01
IGHV4-34	01	01	01
IGHV3-33	01	01	01
IGHV4-31	lost	03	03
IGHV3-30-5	lost	01	01
IGHV4-30-4	lost	lost	01
IGHV3-30-3	03	lost	01/03
IGHV4-30-2	lost	lost	01
IGHV4-30-1	lost	lost	lost
IGHV3-30	04	18	04/18
IGHV4-28	01/05	01	01/05
IGHV2-26	01	01	01
IGHV1-24	01	01	01
IGHV3-23D	01	01	01
IGHV3-23	01	01	01
IGHV3-21	01	01	01
IGHV3-20	01	01	01
IGHV1-18	01	01	01
IGHV3-16	02	02	02
IGHV3-15	01	01	01
IGHV3-13	01	01	01/04
IGHV3-11	01	01	01/05

Continued on next page

Table S9 – *Continued from previous page*

IGHV3-9	01	01	01
IGHV1-8	01	01	01
IGHV5-10-1	lost	lost	01
IGHV3-64D	lost	lost	lost
IGHV3-7	01	01	01
IGHV2-5	02	02	02/Novel
IGHV7-4-1	02	02	lost
IGHV4-4	02/07	01/02	07
IGHV1-3	01	01	02
IGHV1-2	02/04	04/05	02
IGHV6-1	01	01	01

Table S10. IGKV alleles in HG005 trio. The alleles violate the Mendelian rule is highlighted in bold. The alleles without enough parent information are highlighted in italic. "Post-V(D)J" indicates the allele with evidence of V(D)J rearrangement.

Gene	HG005	HG006	HG007
IGKV3D-7	01	01	01
IGKV1D-8	01	01	01/Novel
IGKV1D-43	01	01	01
IGKV1D-42	01	01	01
IGKV3D-11	01/02	01	02
IGKV1D-12	01	01	01
IGKV1D-13	01	01	01
IGKV3D-15	01/Novel	01	01/Novel
IGKV1D-16	01	01	01
IGKV1D-17	01	01	01
IGKV6D-41	01	01	01
IGKV3D-20	01	01	01
IGKV6D-21	02	01/02	02
IGKV2D-24	01	01	01
IGKV2D-26	03/Novel	03/Novel	03/Novel
IGKV2D-28	01	01	01
IGKV2D-29	01/02	01/02	01/02
IGKV2D-30	01	01	01
IGKV1D-33	01	01	01
IGKV1D-37	01	01	01
IGKV1D-39	01	01	01
IGKV2D-40	01	01	01
IGKV2-40	01/01/novel-0	01/01/novel-0	01/01/novel-0
IGKV1-39	01	01/Novel	01
IGKV1-37	01	01	01
IGKV1-33	01	01	01
IGKV2-30	01/02	01/02	01/02
IGKV2-29	01/02	01/02	01/02
IGKV2-28	01/Novel	01/Novel	01/Novel
IGKV1-27	Novel	01/Novel	Novel
IGKV2-24	Novel(I)	01/Novel(I)	Novel(I)/Novel(M)
IGKV6-21	02	01/02	02
IGKV3-20	01	01	01
IGKV1-17	03	01/03	03
IGKV1-16	02	01/02	01/02
IGKV3-15	01	01	01
IGKV1-13	lost	01	lost
IGKV1-12	01	01	01
IGKV3-11	01	01	01
IGKV1-9	Novel	01/Novel	Novel
IGKV1-8	Novel	01/Novel	Novel
IGKV3-7	Novel	04/Novel	Novel
IGKV1-6	01	01	01
IGKV1-5	lost	Novel	Novel
IGKV5-2	Novel	Novel	Novel
IGKV4-1	01	01	01

Table S11. IGLV alleles in HG005 trio. The alleles violate the Mendelian rule is highlighted in bold. The alleles without enough parent information are highlighted in italic. "Post-V(D)J" indicates the allele with evidence of V(D)J rearrangement.

Gene	HG005	HG006	HG007
IGLV4-69	01	01	01
IGLV8-61	01/Novel	01/Novel	01
IGLV4-60	03/Novel	03/Novel	03
IGLV6-57	01/Novel	01/Novel	01/02
IGLV11-55	01/02	02	01/02
IGLV10-54	01	01/02	01
IGLV5-52	01	01	01
IGLV1-51	01	01	01
IGLV1-50	01	01	01/Novel
IGLV9-49	01	01	01
IGLV5-48	01	01/02	01
IGLV1-47	01	01/02	01
IGLV7-46	01/Novel	01/02	Novel
IGLV5-45	02	02/03	02
IGLV1-44	01	01	01
IGLV7-43	01	01/Novel	01
IGLV1-41	02	02	01/02
IGLV1-40	01	01	01
IGLV5-39	01	lost	01
IGLV5-37	01	01	01/Novel
IGLV1-36	01	01	01
IGLV2-33	01	01	01
IGLV3-32	01/Novel	01	01/Novel
IGLV3-27	01	01	01
IGLV3-25	03/Novel(P)	Novel(P)	03/Novel(M)
IGLV2-23	03	03	02/03
IGLV3-22	Novel(M)/Novel(P)	Novel(P)	Novel(M)
IGLV3-21	Novel	Novel	Novel
IGLV3-19	01	01	01
IGLV2-18	02	02	02
IGLV3-16	01/Novel	01/Novel	01
IGLV2-14	Novel	Novel	Novel
IGLV3-12	02	lost	01/02
IGLV2-11	01	01	01
IGLV3-10	01	01	01
IGLV3-9	01	01	01
IGLV2-8	01	01	01
IGLV4-3	01	01	01
IGLV3-1	01	01	01

Table S12. IGHV alleles in HG00733 trio. The alleles violate the Mendelian rule is highlighted in bold. The alleles without enough parent information are highlighted in italic. "Post-V(D)J" indicates the allele with evidence of V(D)J rearrangement. "A.D." is short of "assembly discordance," where the two assemblies carry different alleles for the same gene.

Gene	HG00733-H1	HG00733-H2	HG00731-H1	HG00731-H2	HG00732-H1	HG00732-H2
IGHV7-81	01	01	01	01	01	01
IGHV3-74	01	01	01	01	01	01
IGHV3-73	01	01	01	01	01	02
IGHV3-72	01	01	01	01	01	01
IGHV2-70	15	05	05	15	15	A.D.
IGHV1-69D	*del*	del	del	del	A.D.	01
IGHV1-69-2	*del*	del	del	del	A.D.	01
IGHV2-70D	*del*	del	del	del	A.D.	A.D.
IGHV1-69	04	02	02	04	04	06
IGHV3-66	01	03	03	01	01	03
IGHV3-64	01	02	02	01	01	02
IGHV4-61	Novel(4-59*08)	01	01	Novel(4-59*08)	Novel(4-59*08)	01
IGHV4-59	01	01	01	01	01’	01
IGHV1-58	02	01	01	02	02	01
IGHV3-53	04	01	01	01	04	01
IGHV5-51	01	01	01	01	01	01
IGHV3-49	03	05	05	05	03	05
IGHV3-48	04	02	02	02	04	02
IGHV1-46	01	03	03	01	01	01
IGHV1-45	02	02	02	02	02	02
IGHV3-43	lost	01	01	01	lost	01
IGHV4-39	lost	*01*	Post-VDJ	Post-VDJ	lost	07
IGHV1-38-4	lost	lost	del	lost	lost	01
IGHV3-38-3	lost	lost	del	lost	lost	01
IGHV3-43D	*03*	lost	del	lost	lost	lost
IGHV4-38-2	02	lost	del	lost	02	lost
IGHV3-38	02	lost	02	02	02	02
IGHV3-35	Novel	01	01	01	Novel	01
IGHV4-34	**del**	01	01	01	01	01
IGHV3-33	del	**del**	01	01	del	01
IGHV4-31	del	**del**	01	del	del	del
IGHV3-30-5	del	01	del	del	01	01
IGHV4-30-4	A.D.	01	del	del	01	01
IGHV3-30-3	del	del	del	del	del	01
IGHV4-30-2	del	del	del	del	del	01
IGHV4-30-1	del	del	del	del	del	del
IGHV3-30	18	01	01	del	18	del
IGHV4-28	01	02	02	01	01	02
IGHV2-26	01	01	01	01	01	01
IGHV1-24	01	01	01	01	01	01
IGHV3-23D	01	01	01	del	01	Novel(IGHV3-23*04)
IGHV3-23	01	01	01	01	01	04
IGHV3-21	01	01	01	01	01	01
IGHV3-20	01	*01*	lost	01	01	lost
IGHV1-18	01	*01*	lost	01	01	lost
IGHV3-16	02	02	02	A.D.	02	02
IGHV3-15	01	01	01	01	01	01
IGHV3-13	05	05	05	04	05	01

Continued on next page

Table S12 – *Continued from previous page*

IGHV3-11	06	06	06	05	06	01
IGHV3-9	del	*del*	lost	del	del	01
IGHV1-8	del	*del*	lost	del	del	01
IGHV5-10-1	03	Novel	Novel	01	03	del
IGHV3-64D	*Novel*	06	06	06	A.D.	del
IGHV3-7	*03*	03	03	03	Post-VDJ	01
IGHV2-5	*02*	02	02	02	lost	02
IGHV7-4-1	*01*	*del*	02	02	lost	01
IGHV4-4	*02*	*07*	lost	02	lost	02
IGHV1-3	*Novel(0)*	*Novel(1)*	lost	02	lost	lost
IGHV1-2	*04*	lost	lost	02	lost	lost
IGHV6-1	*01*	lost	lost	01	lost	lost

Table S13. IGKV alleles in HG00733 trio. The alleles violate the Mendelian rule is highlighted in bold. The alleles without enough parent information are highlighted in italic. “Post-V(D)J” indicates the allele with evidence of V(D)J rearrangement. “A.D.” is short of “assembly discordance,” where the two assemblies carry different alleles for the same gene.

Gene	HG00733-H1	HG00733-H2	HG00731-H1	HG00731-H2	HG00732-H1	HG00732-H2
IGKV3D-7	01	lost	01	lost	01	01
IGKV1D-8	01	lost	01	lost	01	02
IGKV1D-43	01	lost	01	lost	01	01
IGKV1D-42	01	*01*	lost	lost	01	02
IGKV3D-11	lost	*01*	lost	lost	01	02
IGKV1D-12	lost	*02*	lost	lost	01	02
IGKV1D-13	lost	*Novel*	lost	lost	01	Novel
IGKV3D-15	lost	*01*	lost	lost	01	03
IGKV1D-16	lost	01	lost	01	01	01
IGKV1D-17	lost	01	lost	01	01	01
IGKV6D-41	lost	01	lost	01	01	01
IGKV3D-20	lost	lost	lost	01	01	lost
IGKV6D-21	lost	lost	lost	02	02	lost
IGKV2D-24	lost	lost	lost	01	01	lost
IGKV2D-26	lost	Novel	lost	Novel	03	lost
IGKV2D-28	lost	01	lost	01	01	lost
IGKV2D-29	lost	01	lost	01	01	lost
IGKV2D-30	lost	01	lost	01	01	lost
IGKV1D-33	lost	01	lost	01	01	lost
IGKV1D-37	lost	01	lost	01	01	lost
IGKV1D-39	lost	01	lost	01	lost	lost
IGKV2D-40	lost	01	lost	01	lost	lost
IGKV2-40	*01*	lost	01	lost	lost	lost
IGKV1-39	*01*	lost	01	lost	lost	lost
IGKV1-37	*01*	lost	01	lost	lost	lost
IGKV1-33	*01*	lost	01	lost	lost	lost
IGKV2-30	*01*	lost	Novel	lost	lost	lost
IGKV2-29	*01*	lost	01	lost	lost	lost
IGKV2-28	*01*	lost	01	lost	lost	lost
IGKV1-27	*01*	lost	01	lost	lost	lost
IGKV2-24	lost	lost	01	lost	lost	lost
IGKV6-21	lost	lost	01	lost	lost	lost
IGKV3-20	lost	lost	01	lost	lost	lost
IGKV1-17	lost	lost	Post-VJ	lost	lost	lost
IGKV1-16	lost	lost	lost	lost	lost	lost
IGKV3-15	lost	lost	lost	lost	lost	lost
IGKV1-13	*01*	lost	lost	lost	lost	lost
IGKV1-12	*01*	lost	lost	lost	lost	lost
IGKV3-11	*01*	lost	lost	lost	lost	lost
IGKV1-9	lost	lost	lost	lost	lost	lost
IGKV1-8	lost	*Novel*	lost	lost	lost	lost
IGKV3-7	lost	*Novel*	lost	lost	lost	lost
IGKV1-6	*01*	*01*	lost	lost	lost	lost
IGKV1-5	*03*	*Novel*	lost	lost	lost	lost
IGKV5-2	*01*	*Post-VJ*	lost	lost	lost	lost
IGKV4-1	*01*	*01*	lost	lost	lost	lost

Table S14. IGLV alleles in HG00733 trio. The alleles violate the Mendelian rule is highlighted in bold. The alleles without enough parent information are highlighted in italic. “Post-V(D)J” indicates the allele with evidence of V(D)J rearrangement. “A.D.” is short of “assembly discordance,” where the two assemblies carry different alleles for the same gene.

Gene	HG00733-H1	HG00733-H2	HG00731-H1	HG00731-H2	HG00732-H1	HG00732-H2
IGLV4-69	01	01	01	01	01	01
IGLV8-61	01	01	01	01	01	01
IGLV4-60	03	02	03	Novel	02	03
IGLV6-57	Novel	01	Novel	02	01	01
IGLV11-55	02	02	02	02	02	02
IGLV10-54	01	01	01	02	01	01
IGLV5-52	01	01	01	01	01	01
IGLV1-51	01	*01*	01	Novel	Post-VJ	01
IGLV1-50	01	*01*	01	01	lost	01
IGLV9-49	01	*01*	01	01	lost	01
IGLV5-48	01	*01*	01	02	lost	02
IGLV1-47	01	*01*	01	01	lost	Novel
IGLV7-46	01	*01*	01	01	lost	01
IGLV5-45	03	*03*	03	02	lost	Novel
IGLV1-44	01	*01*	01	01	lost	01
IGLV7-43	01	*01*	01	01	lost	01
IGLV1-41	02	*Novel*	02	02	lost	02
IGLV1-40	01	*01*	01	01	lost	01
IGLV5-39	Novel	*01*	Novel	del	lost	del
IGLV5-37	Novel	*01*	Novel	01	lost	01
IGLV1-36	01	*01*	01	01	lost	01
IGLV2-33	01	*01*	01	01	lost	01
IGLV3-32	01	*01*	01	01	lost	01
IGLV3-27	01	*01*	01	01	lost	01
IGLV3-25	03	*03*	03	02	lost	03
IGLV2-23	02	*02*	02	03	lost	03
IGLV3-22	01	*01*	01	Novel	lost	01
IGLV3-21	02	*Novel*	02	02	lost	03
IGLV3-19	01	*01*	01	01	lost	01
IGLV2-18	01	*02*	01	02	lost	02
IGLV3-16	01	*01*	01	01	lost	01
IGLV2-14	01	*01*	01	04	lost	04
IGLV3-12	02	*02*	02	02	lost	Novel
IGLV2-11	01	*01*	01	01	lost	01
IGLV3-10	01	*01*	01	Novel	lost	01
IGLV3-9	01	*01*	01	01	lost	01
IGLV2-8	01	*01*	01	01	lost	03
IGLV4-3	01	*01*	01	01	lost	01
IGLV3-1	01	*01*	01	01	lost	01

Table S15. IGHV alleles in NA19240 trio. The alleles violate the Mendelian rule is highlighted in bold. The alleles without enough parent information are highlighted in italic. “Post-V(D)J” indicates the allele with evidence of V(D)J rearrangement. “A.D.” is short of “assembly discordance,” where the two assemblies carry different alleles for the same gene.

Gene	NA19240-H1	NA19240-H2	NA9239-H1	NA19239-H2	NA19238-H1	NA19238-H2
IGHV7-81	Novel(0)	01	01	01	Novel(0)	Novel(1)
IGHV3-74	01	01	01	01	01	01
IGHV3-73	02	02	02	01	02	02
IGHV3-72	01	01	01	01	01	01
IGHV2-70	A.D.	**19**	Novel	15	Novel	04
IGHV1-69D	*del*	**del**	Novel(IGHV1-69*05)	del	lost	del
IGHV1-69-2	*del*	**del**	01	del	lost	del
IGHV2-70D	*del*	**del**	14	del	lost	del
IGHV1-69	12	**05**	14	14	12	A.D.
IGHV3-66	02	03	03	Novel(P)	02	Novel(M)
IGHV3-64	07	01	01	07	07	01
IGHV4-61	09	02	02	09	09	Novel(IGHV4-59*08)
IGHV4-59	11	01	01	Novel	11	01
IGHV1-58	02	02	02	Novel	02	02
IGHV3-53	04	02	02	04	04	Post-VDJ
IGHV5-51	01	03	03	01	01	lost
IGHV3-49	03	03	03	03	03	lost
IGHV3-48	01	01	01	04	01	lost
IGHV1-46	01	03	03	01	01	lost
IGHV1-45	02	02	02	02	02	lost
IGHV3-43	01	01	01	01	01	lost
IGHV4-39	01	01	01	07	01	lost
IGHV1-38-4	01	01	01	01	01	lost
IGHV3-38-3	01	Novel	Novel	01	01	lost
IGHV3-43D	A.D.	A.D.	Novel	lost	03	lost
IGHV4-38-2	02	01	01	02	02	lost
IGHV3-38	03	02	02	03	03	lost
IGHV3-35	01	01	01	01	01	lost
IGHV4-34	01	01	01	01	01	lost
IGHV3-33	*del*	06	06	del	lost	lost
IGHV4-31	*del*	03	03	del	lost	lost
IGHV3-30-5	A.D.	del	del	del	lost	lost
IGHV4-30-4	*del*	del	del	del	lost	lost
IGHV3-30-3	**del**	*del*	03	del	lost	lost
IGHV4-30-2	**del**	*del*	01	del	lost	lost
IGHV4-30-1	del	*del*	del	del	lost	lost
IGHV3-30	18	*del*	18	A.D.	lost	lost
IGHV4-28	07	*del*	07	01	lost	lost
IGHV2-26	01	*01*	01	Novel	lost	lost
IGHV1-24	01	*01*	01	01	lost	lost
IGHV3-23D	01	*del*	01	del	lost	lost
IGHV3-23	lost	01	04	01	lost	lost
IGHV3-21	lost	03	03	01	lost	lost
IGHV3-20	lost	04	04	lost	lost	lost
IGHV1-18	lost	01	01	lost	lost	lost
IGHV3-16	*02*	02	02	02	lost	lost
IGHV3-15	*01*	01	01	01	lost	lost
IGHV3-13	*01*	03	03	01	lost	lost

Continued on next page

Table S15 – *Continued from previous page*

IGHV3-11	*04*	04	04	05	lost	lost
IGHV3-9	Post-VDJ	Post-VDJ	03	del	lost	lost
IGHV1-8	lost	lost	03	del	lost	lost
IGHV5-10-1	lost	lost	del	01	lost	lost
IGHV3-64D	lost	lost	del	Novel	lost	lost
IGHV3-7	*01*	lost	01	01	lost	lost
IGHV2-5	lost	lost	02	02	lost	lost
IGHV7-4-1	*02*	lost	01	02	lost	lost
IGHV4-4	*08*	lost	A.D.	08	lost	lost
IGHV1-3	*03*	lost	01	03	lost	lost
IGHV1-2	lost	lost	04	02	lost	lost
IGHV6-1	lost	lost	01	01	lost	lost

Table S16. IGKV alleles in NA19240 trio. The alleles violate the Mendelian rule is highlighted in bold. The alleles without enough parent information are highlighted in italic. "Post-V(D)J" indicates the allele with evidence of V(D)J rearrangement. "A.D." is short of "assembly discordance," where the two assemblies carry different alleles for the same gene.

Gene	NA19240-H1	NA19240-H2	NA9239-H1	NA19239-H2	NA19238-H1	NA19238-H2
IGKV3D-7	lost	*01*	01	lost	01	01
IGKV1D-8	lost	*02*	02	lost	01	02
IGKV1D-43	lost	*01*	lost	lost	01	01
IGKV1D-42	lost	*01*	01	lost	01	02
IGKV3D-11	lost	*01*	02	lost	01	02
IGKV1D-12	lost	*Novel(IGKV1-12*01)*	02	lost	01	02
IGKV1D-13	lost	*01*	02	lost	01	02
IGKV3D-15	lost	*01*	03	lost	lost	01
IGKV1D-16	lost	*01*	01	lost	lost	01
IGKV1D-17	lost	*01*	01	lost	lost	A.D.
IGKV6D-41	lost	lost	01	lost	lost	01
IGKV3D-20	lost	lost	lost	lost	01	lost
IGKV6D-21	lost	lost	lost	lost	02	lost
IGKV2D-24	lost	lost	lost	lost	01	lost
IGKV2D-26	lost	lost	lost	lost	A.D.	lost
IGKV2D-28	lost	lost	lost	lost	01	lost
IGKV2D-29	lost	lost	lost	lost	A.D.	lost
IGKV2D-30	lost	lost	lost	lost	01	lost
IGKV1D-33	lost	lost	lost	lost	01	lost
IGKV1D-37	lost	lost	lost	lost	01	lost
IGKV1D-39	lost	lost	lost	lost	01	lost
IGKV2D-40	lost	lost	lost	lost	lost	lost
IGKV2-40	lost	lost	lost	lost	lost	02
IGKV1-39	lost	lost	lost	02	lost	02
IGKV1-37	lost	lost	lost	01	lost	01
IGKV1-33	lost	lost	lost	01	lost	01
IGKV2-30	lost	lost	lost	lost	lost	01
IGKV2-29	lost	lost	lost	lost	lost	01
IGKV2-28	lost	lost	lost	lost	lost	01
IGKV1-27	lost	lost	lost	lost	lost	01
IGKV2-24	lost	lost	lost	lost	lost	01
IGKV6-21	lost	lost	lost	lost	lost	02
IGKV3-20	lost	lost	lost	lost	lost	lost
IGKV1-17	lost	lost	lost	lost	lost	lost
IGKV1-16	lost	lost	lost	lost	lost	lost
IGKV3-15	lost	lost	lost	lost	lost	lost
IGKV1-13	*01*	lost	lost	lost	lost	lost
IGKV1-12	*01*	lost	lost	lost	lost	lost
IGKV3-11	01	lost	lost	01	01	lost
IGKV1-9	01	lost	lost	01	01	lost
IGKV1-8	01	lost	lost	01	01	lost
IGKV3-7	04	lost	lost	04	04	lost
IGKV1-6	01	lost	lost	01	01	lost
IGKV1-5	03	lost	lost	03	03	lost
IGKV5-2	01	lost	lost	lost	01	lost
IGKV4-1	lost	lost	lost	lost	Novel	lost

Table S17. IGLV alleles in NA19240 trio. The alleles violate the Mendelian rule is highlighted in bold. The alleles without enough parent information are highlighted in italic. "Post-V(D)J" indicates the allele with evidence of V(D)J rearrangement. "A.D." is short of "assembly discordance," where the two assemblies carry different alleles for the same gene.

Gene	NA19240-H1	NA19240-H2	NA9239-H1	NA19239-H2	NA19238-H1	NA19238-H2
IGLV4-69	01	01	01	01	01	01
IGLV8-61	Novel	01	01	01	Novel	01
IGLV4-60	03	03	03	03	03	Novel
IGLV6-57	Novel	01	01	01	Novel	01
IGLV11-55	01	02	02	02	01	02
IGLV10-54	02	01	01	02	02	02
IGLV5-52	01	01	01	01	01	01
IGLV1-51	02	Novel	Novel	02	02	01
IGLV1-50	01	01	01	01	01	01
IGLV9-49	01	01	01	01	01	01
IGLV5-48	01	01	01	01	01	01
IGLV1-47	01	01	01	01	01	Novel
IGLV7-46	Novel	01	01	01	Novel	01
IGLV5-45	Novel	02	02	01	Novel	01
IGLV1-44	Novel	01	01	01	Novel	01
IGLV7-43	01	01	01	01	01	01
IGLV1-41	Novel	01	01	Novel	Novel	02
IGLV1-40	01	01	01	01	01	01
IGLV5-39	01	01	01	del	01	del
IGLV5-37	Novel	Novel	Novel	01	Novel	01
IGLV1-36	01	Novel(P)	Novel(P)	01	01	Novel(M)
IGLV2-33	01	01	01	01	01	01
IGLV3-32	01	01	01	01	01	01
IGLV3-27	01	01	01	01	01	01
IGLV3-25	03	03	03	03	03	03
IGLV2-23	03	02	02	03	03	02
IGLV3-22	Novel(I)	Novel(P)	Novel(P)	Novel(I)	Novel(I)	01
IGLV3-21	Novel	03	03	Novel	Novel	Novel
IGLV3-19	01	01	01	01	01	01
IGLV2-18	02	02	02	02	02	02
IGLV3-16	Novel(I)	Novel(I)	Novel(I)	Novel(I)	Novel(I)	Novel(M)
IGLV2-14	01	01	01	01	01	Novel
IGLV3-12	02	02	02	02	02	02
IGLV2-11	01	01	01	01	01	01
IGLV3-10	01	01	01	01	01	01
IGLV3-9	01	01	01	01	01	01
IGLV2-8	01	01	01	01	01	01
IGLV4-3	01	01	01	01	01	01
IGLV3-1	01	A.D.	01	01	01	01

Table S18. IGHV alleles in HG00514 trio. The alleles violate the Mendelian rule is highlighted in bold. The alleles without enough parent information are highlighted in italic. "Post-V(D)J" indicates the allele with evidence of V(D)J rearrangement. "A.D." is short of "assembly discordance," where the two assemblies carry different alleles for the same gene.

Gene	HG00514-H1	HG00514-H2	HG00512-H1	HG00512-H2	HG00513-H1	HG00513-H2
IGHV7-81	01	01	01	01	01	01
IGHV3-74	01	01	01	01	01	01
IGHV3-73	02	01	02	01	02	02
IGHV3-72	01	01	01	01	01	01
IGHV2-70	*01*	05	01	05	Novel	Post-VDJ
IGHV1-69D	del	del	del	del	del	del
IGHV1-69-2	del	del	del	del	del	del
IGHV2-70D	del	del	del	del	del	del
IGHV1-69	01	02	01	02	04	01
IGHV3-66	03	02	03	02	01	03
IGHV3-64	01	01	02	01	01	01
IGHV4-61	02	02	01	02	IGHV4-59*08	02
IGHV4-59	01	01	01	01	01	01
IGHV1-58	02	02	01	02	02	02
IGHV3-53	01	04	01	04	04	01
IGHV5-51	Novel	Post-VDJ	01	01	01	Novel
IGHV3-49	03	**03**	05	04	03	03
IGHV3-48	02	02	02	03	04	02
IGHV1-46	01	01	01	01	01	01
IGHV1-45	02	02	02	03	02	02
IGHV3-43	01	01	01	02	01	01
IGHV4-39	01	01	01	01	07	01
IGHV1-38-4	del	del	del	del	01	del
IGHV3-38-3	del	del	del	del	01	del
IGHV3-43D	del	del	del	del	03	del
IGHV4-38-2	del	del	del	del	02	del
IGHV3-38	02	02	02	02	02	02
IGHV3-35	01	01	01	01	Novel	01
IGHV4-34	01	01	01	01	01	01
IGHV3-33	01	01	del	01	06	01
IGHV4-31	del	del	del	03	03	del
IGHV3-30-5	del	del	del	01	del	del
IGHV4-30-4	del	del	del	del	del	del
IGHV3-30-3	del	del	del	del	del	del
IGHV4-30-2	del	del	del	del	del	del
IGHV4-30-1	del	del	del	del	del	del
IGHV3-30	del	**del**	Novel	del	A.D.	del
IGHV4-28	01	01	01	01	05	01
IGHV2-26	Novel(M)	**Novel(M)**	01	01	A.D.	Novel(M)
IGHV1-24	01	01	01	01	01	01
IGHV3-23D	del	del	del	01	del	del
IGHV3-23	01	01	01	01	01	01
IGHV3-21	01	01	01	01	01	01
IGHV3-20	01	lost	01	04	01	01
IGHV1-18	01	lost	lost	01	01	01
IGHV3-16	02	lost	02	Novel	02	02
IGHV3-15	01	lost	01	01	01	01
IGHV3-13	04	lost	05	04	04	lost

Continued on next page

Table S18 – *Continued from previous page*

IGHV3-11	05	lost	06	05	05	lost
IGHV3-9	del	lost	del	del	01	del
IGHV1-8	del	lost	del	del	01	del
IGHV5-10-1	01	lost	03	01	del	01
IGHV3-64D	Novel	lost	06	Novel	del	Novel
IGHV3-7	Post-VDJ	lost	03	Novel	01	01
IGHV2-5	lost	lost	Post-VDJ	01	02	02
IGHV7-4-1	lost	lost	lost	del	02	02
IGHV4-4	lost	lost	lost	07	01	07
IGHV1-3	lost	lost	lost	02	01	02
IGHV1-2	lost	lost	lost	02	05	05
IGHV6-1	lost	lost	lost	01	01	01

Table S19. IGKV alleles in HG00514 trio. The alleles violate the Mendelian rule is highlighted in bold. The alleles without enough parent information are highlighted in italic. "Post-V(D)J" indicates the allele with evidence of V(D)J rearrangement. "A.D." is short of "assembly discordance," where the two assemblies carry different alleles for the same gene.

Gene	HG00514-H1	HG00514-H2	HG00512-H1	HG00512-H2	HG00513-H1	HG00513-H2
IGKV3D-7	lost	01	01	lost	lost	01
IGKV1D-8	lost	01	01	lost	lost	del
IGKV1D-43	lost	01	01	lost	01	del
IGKV1D-42	lost	01	01	lost	01	01
IGKV3D-11	lost	01	01	lost	Novel(IGKV3-11)	01
IGKV1D-12	lost	01	01	lost	01	01
IGKV1D-13	lost	01	01	lost	01	01
IGKV3D-15	lost	01	01	lost	01	01
IGKV1D-16	lost	01	01	lost	01	01
IGKV1D-17	lost	01	01	lost	01	01
IGKV6D-41	lost	01	01	lost	01	01
IGKV3D-20	lost	01	01	lost	lost	01
IGKV6D-21	lost	02	02	lost	lost	02
IGKV2D-24	lost	01	01	lost	lost	01
IGKV2D-26	lost	03	03	lost	lost	03
IGKV2D-28	lost	01	01	lost	lost	01
IGKV2D-29	lost	01	01	lost	lost	02
IGKV2D-30	lost	01	01	lost	lost	01
IGKV1D-33	lost	01	01	lost	lost	01
IGKV1D-37	lost	01	01	lost	lost	01
IGKV1D-39	lost	A.D.	01	lost	lost	01
IGKV2D-40	lost	01	01	lost	lost	01
IGKV2-40	01	lost	lost	01	01	Post-VJ
IGKV1-39	01	lost	lost	01	01	lost
IGKV1-37	01	lost	lost	01	01	lost
IGKV1-33	01	lost	lost	01	01	lost
IGKV2-30	*01*	lost	lost	02	Post-VJ	lost
IGKV2-29	*01*	lost	lost	02	lost	lost
IGKV2-28	*01*	lost	lost	01	lost	lost
IGKV1-27	*01*	lost	lost	Novel	lost	lost
IGKV2-24	*01*	lost	lost	01	lost	lost
IGKV6-21	*01*	lost	lost	01	lost	lost
IGKV3-20	*01*	lost	lost	01	lost	lost
IGKV1-17	*01*	lost	lost	01	lost	lost
IGKV1-16	*02*	lost	lost	02	lost	lost
IGKV3-15	*01*	lost	lost	01	lost	lost
IGKV1-13	*01*	lost	lost	01	lost	lost
IGKV1-12	*01*	lost	lost	01	lost	lost
IGKV3-11	*01*	lost	lost	01	lost	lost
IGKV1-9	*01*	lost	lost	01	lost	lost
IGKV1-8	*01*	lost	lost	01	lost	lost
IGKV3-7	*04*	lost	lost	04	lost	lost
IGKV1-6	*01*	lost	lost	01	lost	lost
IGKV1-5	*03*	lost	lost	01	lost	lost
IGKV5-2	*01*	lost	lost	Novel	lost	lost
IGKV4-1	*Novel*	lost	lost	Novel(P)	lost	lost

Table S20. IGLV alleles in HG00514 trio. The alleles violate the Mendelian rule is highlighted in bold. The alleles without enough parent information are highlighted in italic. "Post-V(D)J" indicates the allele with evidence of V(D)J rearrangement. "A.D." is short of "assembly discordance," where the two assemblies carry different alleles for the same gene.

Gene	HG00514-H1	HG00514-H2	HG00512-H1	HG00512-H2	HG00513-H1	HG00513-H2
IGLV4-69	01	01	01	01	01	Novel
IGLV8-61	02	02	02	01	02	01
IGLV4-60	Novel	03	03	03	Novel	03
IGLV6-57	Novel(I)	Novel(P)	Novel(P)	Novel(I)	Novel(I)	01
IGLV11-55	02	01	01	02	02	02
IGLV10-54	01	01	01	01	01	02
IGLV5-52	01	01	01	01	01	01
IGLV1-51	01	01	01	01	01	01
IGLV1-50	01	01	01	01	01	01
IGLV9-49	01	01	01	01	01	01
IGLV5-48	01	01	01	01	01	01
IGLV1-47	01	01	01	01	01	01
IGLV7-46	01	01	01	Novel	01	Novel
IGLV5-45	03	03	03	02	03	02
IGLV1-44	01	01	01	01	01	01
IGLV7-43	01	01	01	01	01	01
IGLV1-41	Novel	02	02	02	Novel	01
IGLV1-40	01	01	01	01	01	01
IGLV5-39	del	01	01	01	del	del
IGLV5-37	01	01	01	01	01	01
IGLV1-36	01	01	01	01	01	01
IGLV2-33	01	01	01	01	01	01
IGLV3-32	01	01	01	01	01	01
IGLV3-27	01	01	01	01	01	01
IGLV3-25	Novel	03	03	03	Novel	Novel
IGLV2-23	03	03	03	02	03	03
IGLV3-22	01	01	01	01	01	01
IGLV3-21	Novel	Novel	Novel	Novel	Novel	Novel
IGLV3-19	01	01	01	01	01	01
IGLV2-18	02	Novel	Novel	02	02	02
IGLV3-16	01	01	01	01	01	01
IGLV2-14	Novel(M)	Novel(P)	Novel(P)	04	Novel(M)	03
IGLV3-12	01	01	01	Novel	01	02
IGLV2-11	01	01	01	01	01	01
IGLV3-10	01	01	01	01	01	01
IGLV3-9	01	01	01	01	01	01
IGLV2-8	01	01	01	01	01	01
IGLV4-3	01	01	01	01	01	01
IGLV3-1	**Novel(0)**	**Novel(1)**	01	01	01	01

8 The flanking sequences where gAIRR-seq and gAIRR-call disagree personal assembly

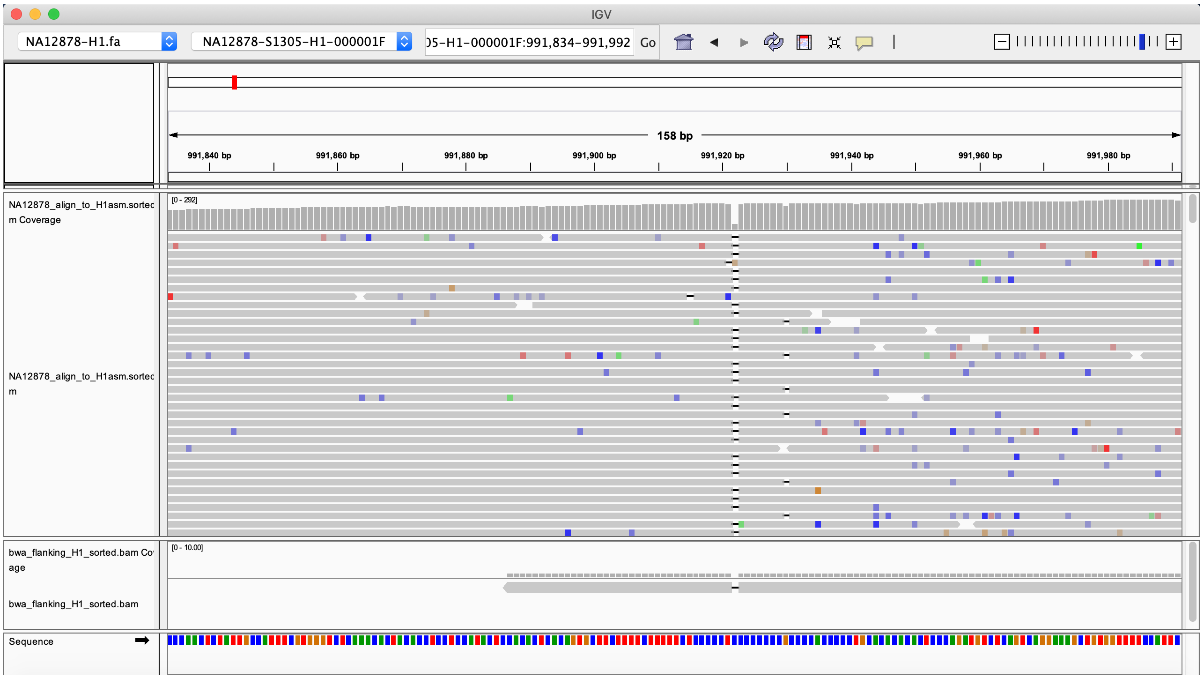

Fig. S6. The Integrated Genomics Viewer[45] visualization of the position where gAIRR-seq and gAIRR-call disagree with the HG001 assembly[31]. The reference is the H1 of the assembly. The aligned sequences from top to bottom are gAIRR-seq reads and gAIRR-called flanking sequences aligned to the assembly. The disagreement at position 991922 is homopolymers C where the assembly is 8 consecutive guanines while gAIRR-seq and gAIRR-call support 7 consecutive guanines.

There are three extended alleles not aligned perfectly to the personal assemblies HG001 and HG002, but we still considered them as true positives after manual inspection. The genes *TRBV20-1* in HG001-H1, *TRBV17* in HG002-maternal, and *TRBV20-1* in HG002-paternal all carried one indel with respect to the personal assemblies. All three indels were in homopolymeric regions, which were known to be error-prone using PacBio sequencing. For example, in *TRBV17* the called allele had 7 consecutive guanines in the flanking region, while the assembly had 8 consecutive guanines (Supplementary Fig. S6 We examined these regions with the deep-coverage read sets sequenced with gAIRR-seq using the Integrated Genomics Viewer[45] and concluded the called alleles were likely to be correct.

9 gAIRR-annotate results on collected samples

Table S21. Number of TR alleles for HQ-12 set (from 12 samples with 58 haplotypes). The numbers of annotated novel alleles and total alleles are shown in columns *#novel* and *#total* respectively.

	TRV		TRD (+hep)		TRJ	
haplotype	#novel	#total	#novel	#total	#novel	#total
HG001-H1[31]	14	145	0	5	2	84
HG001-H2[31]	19	146	0	5	3	84
HG001-CCS-H1[32]	20	145	0	5	3	84
HG001-CCS-H2[32]	11	144	0	5	2	84
HG002-P[31]	23	144	0	2	4	39
HG002-M[31]	17	147	0	5	3	84
HG002-CCS-Canu-P[33]	24	144	0	2	4	39
HG002-CCS-Canu-M[33]	19	148	0	5	3	84
HG002-CCS-Falcon-P[33]	20	144	0	2	4	39
HG002-CCS-Falcon-M[33]	21	146	0	5	4	84
HG002-CCS-wtdbg2-P[33]	23	142	0	2	4	39
HG002-CCS-wtdbg2-M[33]	18	145	0	5	3	82
HG002-hifiasm-P[34]	23	144	0	2	4	77
HG002-hifiasm-M[34]	17	147	0	5	3	84
HG002-CCS-H1[32]	21	145	0	2	5	75
HG002-CCS-H2[32]	19	147	0	5	3	84
HG00731-CCS-H1[32]	12	143	0	5	3	84
HG00731-CCS-H2[32]	8	144	0	5	2	84
HG00731-CLR-H1[32]	10	144	0	5	3	84
HG00731-CLR-H2[32]	12	145	0	5	2	84
HG00732-CCS-H1[32]	16	147	0	5	1	84
HG00732-CCS-H2[32]	11	145	0	5	0	84
HG00732-CLR-H1[32]	16	147	0	5	1	84
HG00732-CLR-H2[32]	14	146	0	5	0	84
HG00733-hifiasm-H1[34]	13	143	0	5	3	84
HG00733-hifiasm-H2[34]	11	145	0	5	0	84
HG00733-HiFi-v0-H1[35]	13	143	0	5	3	84
HG00733-HiFi-v0-H2[35]	11	145	0	5	0	84
HG00733-CCS-H1[32]	11	145	0	5	0	84
HG00733-CCS-H2[32]	13	143	0	5	3	84
HG00733-CLR-H1[32]	12	145	0	5	3	84
HG00733-CLR-H2[32]	14	145	0	5	0	84
HG00512-CCS-H1[32]	10	141	0	5	3	84
HG00512-CCS-H2[32]	20	144	0	5	5	84
HG00512-CLR-H1[32]	16	141	0	5	5	84
HG00512-CLR-H2[32]	16	144	0	5	3	84
HG00513-CCS-H1[32]	11	144	0	5	3	84
HG00513-CCS-H2[32]	15	145	0	5	3	84
HG00513-CLR-H1[32]	13	138	0	5	3	84
HG00513-CLR-H2[32]	16	138	0	5	3	84
HG00514-CCS-H1[32]	11	149	0	5	3	84
HG00514-CCS-H2[32]	15	144	0	5	3	84
HG00514-CLR-H1[32]	17	146	0	5	3	84
HG00514-CLR-H2[32]	23	144	0	5	4	84
NA19238-CCS-H1[32]	32	143	0	5	2	84

Continued on next page

Table S21 – *Continued from previous page*

NA19238-CCS-H2[32]	24	145	0	5	3	84
NA19238-CLR-H1[32]	34	156	0	5	2	84
NA19238-CLR-H2[32]	30	149	0	5	3	84
NA19239-CCS-H1[32]	24	144	0	5	2	84
NA19239-CCS-H2[32]	23	144	0	5	4	84
NA19239-CLR-H1[32]	22	144	0	5	2	84
NA19239-CLR-H2[32]	25	144	0	5	4	84
NA19240-CCS-H1[32]	23	144	0	5	2	84
NA19240-CCS-H2[32]	22	145	0	5	5	84
NA19240-CLR-H1[32]	24	144	0	5	2	84
NA19240-CLR-H2[32]	23	153	0	5	5	84
CHM13-hifiasm[34]	11	147	0	5	1	86
CHM13-T2T[36]	11	147	0	5	1	86

Table S22. Number of TR alleles for HQ-12 sample's consensus. An allele sequence is considered positive only if the allele is called from more than half of the assemblies.

	TRV		TRD (+hep)		TRJ	
sample's consensus	#novel	#total	#novel	#total	#novel	#total
HG001	29	188	0	5	4	87
HG002	31	186	0	5	6	89
HG00731	17	174	0	5	4	89
HG00732	22	173	0	5	1	88
HG00733	21	183	0	6	3	81
HG00512	26	183	0	6	6	89
HG00513	18	156	0	5	5	88
HG00514	19	158	0	5	4	88
NA19238	47	198	0	5	5	91
NA19239	39	190	0	5	5	91
NA19240	42	192	0	5	6	92
CHM13	11	147	0	5	1	85

Table S23. Number of TR alleles for the additional-24 assemblies[32].

	TRV		TRD (+hep)		TRJ	
haplotype	#novel	#total	#novel	#total	#novel	#total
HG00096-CLR-H1	22	144	0	5	2	84
HG00096-CLR-H2	12	143	0	5	1	84
HG00171-CLR-H1	13	144	0	5	2	84
HG00171-CLR-H2	17	147	0	5	2	84
HG00864-CLR-H1	22	150	0	5	2	84
HG00864-CLR-H2	21	150	0	5	4	84
HG01114-CLR-H1	23	147	0	5	0	84
HG01114-CLR-H2	18	150	0	5	1	84
HG01505-CLR-H1	21	144	0	5	2	84
HG01505-CLR-H2	17	142	0	5	1	84
HG01596-CLR-H1	21	145	0	5	3	84
HG01596-CLR-H2	22	151	0	5	1	84
HG02011-CLR-H1	26	144	0	5	2	83
HG02011-CLR-H2	12	144	0	5	1	84
HG02492-CLR-H1	22	144	0	5	1	82
HG02492-CLR-H2	15	152	0	5	3	84
HG02587-CLR-H1	21	145	0	5	2	84
HG02587-CLR-H2	28	142	0	5	3	84
HG02818-CCS-H1	28	145	0	5	1	84
HG02818-CCS-H2	23	145	0	5	3	84
HG03009-CLR-H1	21	150	0	4	1	84
HG03009-CLR-H2	17	144	0	5	1	84
HG03065-CLR-H1	24	147	0	5	2	84
HG03065-CLR-H2	31	147	0	5	3	84
HG03125-CCS-H1	24	144	0	5	2	84
HG03125-CCS-H2	22	147	0	5	3	84
HG03371-CLR-H1	27	145	0	5	4	84
HG03371-CLR-H2	29	142	0	5	1	84
HG03486-CCS-H1	19	145	0	5	5	84
HG03486-CCS-H2	24	145	0	5	3	84
HG03683-CLR-H1	15	144	0	5	1	84
HG03683-CLR-H2	19	144	0	5	2	84
HG03732-CLR-H1	10	144	0	5	3	84
HG03732-CLR-H2	23	144	0	5	2	84
NA12329-CLR-H1	9	145	0	5	1	84
NA12329-CLR-H2	11	144	0	5	2	84
NA18534-CLR-H1	26	144	0	5	3	84
NA18534-CLR-H2	15	144	0	5	3	84
NA18939-CLR-H1	19	144	0	5	3	84
NA18939-CLR-H2	11	144	0	5	5	84
NA19650-CLR-H1	23	147	0	5	1	84
NA19650-CLR-H2	20	141	0	5	3	84
NA19983-CLR-H1	20	145	0	5	2	84
NA19983-CLR-H2	29	144	0	5	2	84
NA20509-CLR-H1	12	146	0	5	2	84
NA20509-CLR-H2	18	142	0	5	0	84
NA20847-CLR-H1	17	144	0	5	0	84
NA20847-CLR-H2	17	141	0	5	2	92*

*: NA20847-CLR-H2 seems to have more TRJ alleles than other haplotypes. In gAIRR-annotate details, the assembly

NA20847-CLR-H2 has two contigs covering the same TRGJ gene locus. It is either due to NA20847 having extra TRGJ genes or misassembly in the region.

10 TRA/TRD locus representation and version difference of IMGT

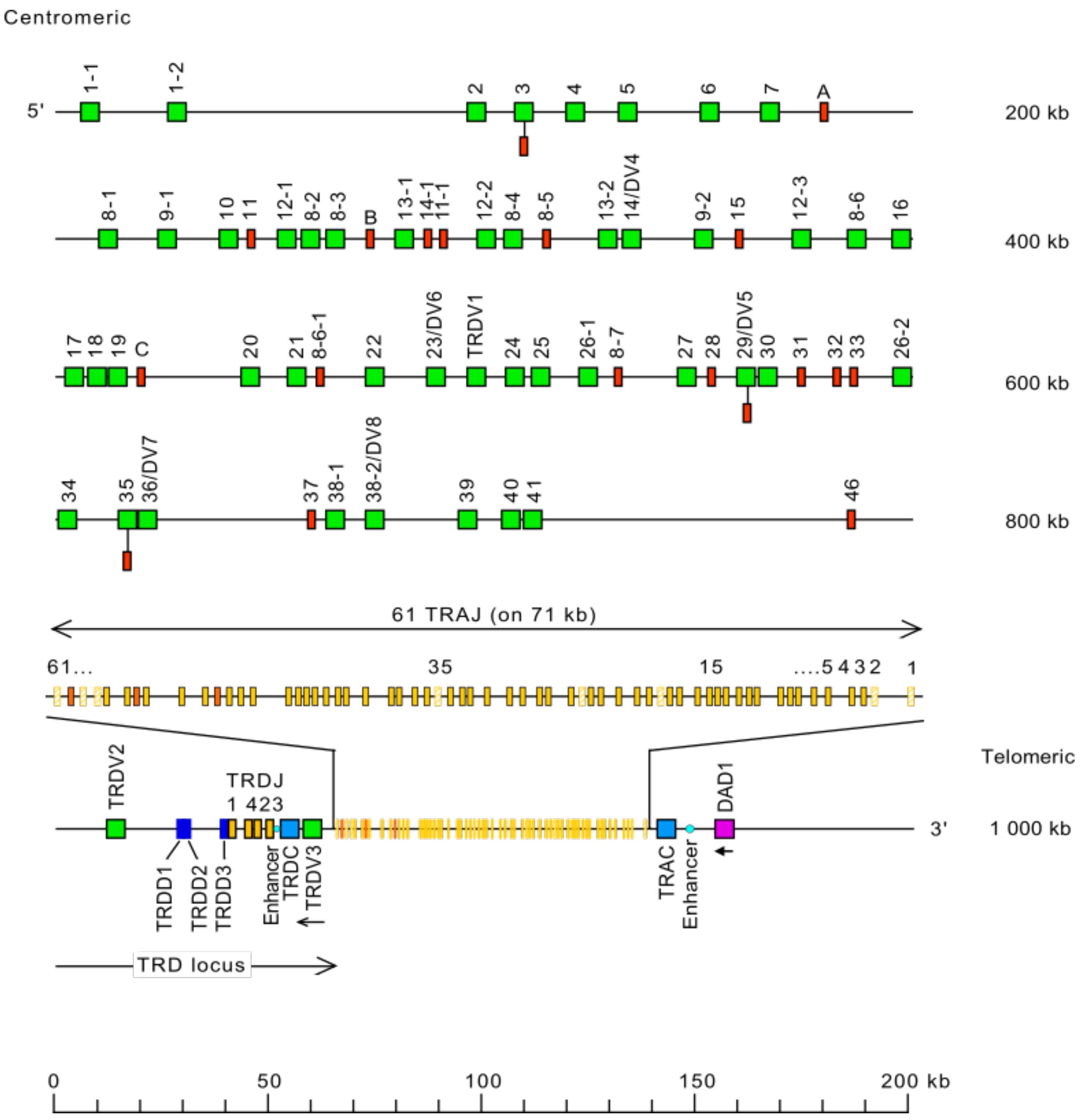

Fig. S7. The locus representation of TRA/TRD alleles downloaded from IMGT[38]

There are some pseudogenes, in red, shown in the IMGT locus representation but not found by gAIRR-annotate. It is because the alleles information used by gAIRR-annotate and gAIRR-call is collected from IMGT version 3.1.22 (2019-04-03), while the representation is from the latest IMGT version v3.1.33 (2021-03-22) by the completion of this manuscript. In version 3.1.22, some TR pseudogenes have not been discovered yet. gAIRR-annotate cannot detect genes that are not in the database, so the latest found pseudogenes are missing in the gAIRR-annotate result. In other words, gAIRR-annotate can only detect novel alleles but not novel genes. After the updates from 3.1.22 to 3.1.33, there are 26 more human TRV alleles and three more human TRJ alleles. In the 26 novel alleles updated by the IMGT, 11 are alleles from 8 novel genes (all pseudogenes), which are not considered by gAIRR-annotate.

11 TRG germline variation of HG001 concordant with IMGT

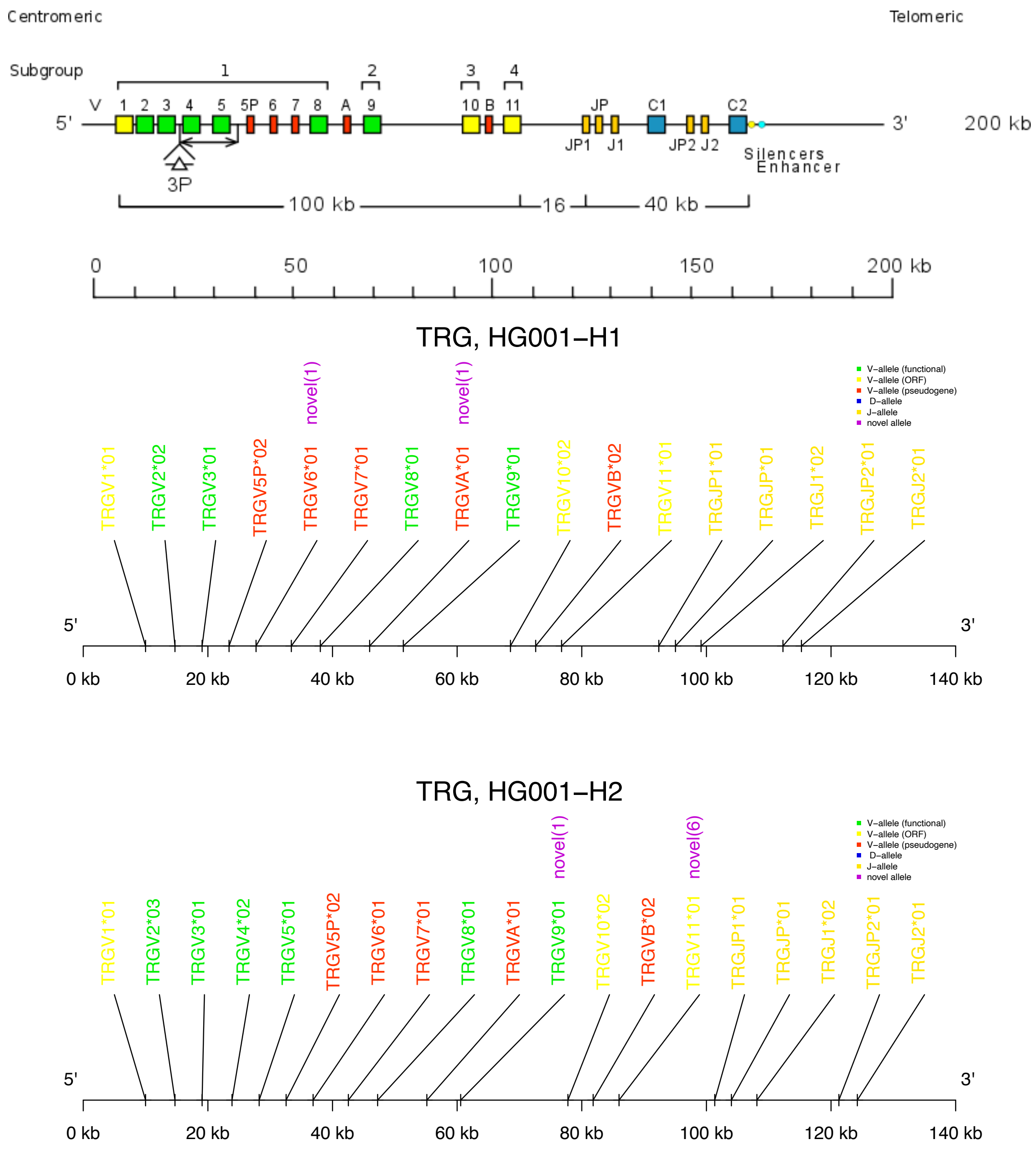

Fig. S8. Subfigures from top to bottom are the locus representation of TRG alleles from IMGT[38], gAIRR-annotate results of HG001-H1, and results of HG001-H2. The difference of H1 and H2 in *TRGV4* and *TRGV5* are recorded as germline variants in IMGT.

12 The difference of read numbers in the deletion of HG002 chromosome 14 related to TRA/TRD alleles

Table S24. Number of reads aligned inside (centromeric region) and outside (telomeric region) the deletion region. Position 22,982,924 is the 3’ side boundary of the deletion. The centromeric region is defined as the position 22,979,200-22,982,924. The telomeric region is defined as the position 22,982,924-22,986,400.

Sample	centromeric region read #	telomeric region read #	drop-off rate
HG002 (son)	944	1599	0.622
HG003 (father)	1701	1692	1.005
HG004 (mother)	2274	1999	1.138

13 HG002's deletion on chromosome 7

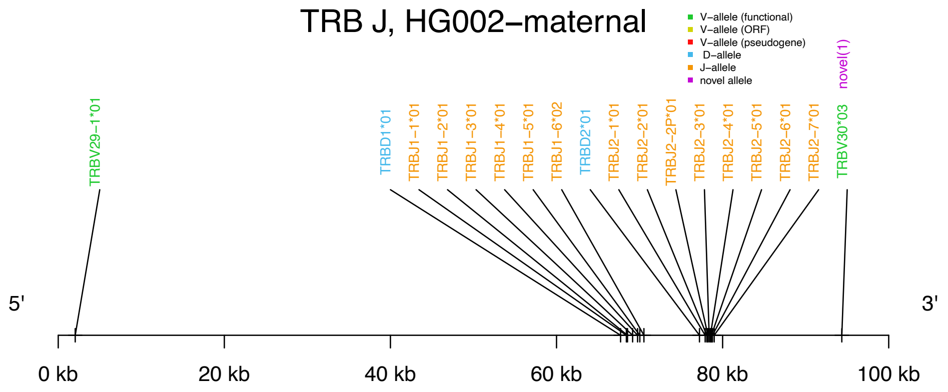

(a)

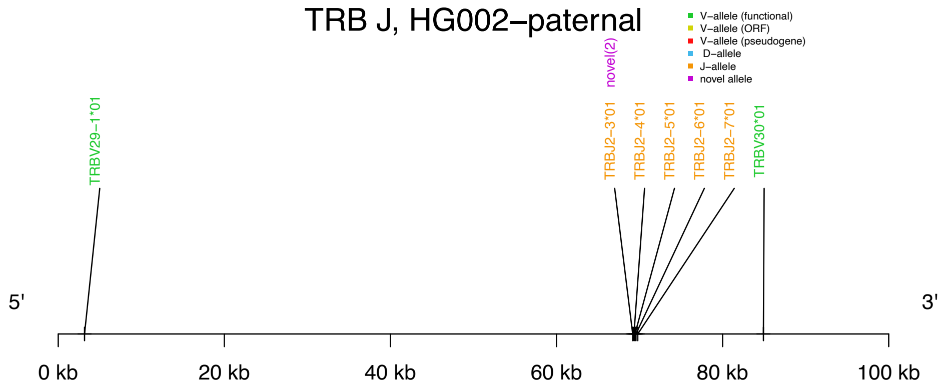

(b)

Fig. S9. The 10 kbp structural variation in the TRB J region of HG002. (a) maternal haplotype, (b) paternal haplotype.

We identified a 10 kb deletion using gAIRR-annotate at the TRB locus on chromosome 7q34 for HG002. We aligned the gAIRR-seqed reads of the HG002 trio to GRCh38 (Supplementary Fig. S10 (a)). There was a noticeable read depth drop in the region in between the red arrows (chr7:142,786,222-142,796,848). The abrupt read depth changes and the mate-pair aligns stretching long distances indicated a deletion in one of the HG002's haplotype. It is worth mentioning that a blue arrow indicates an SNP in HG002 in Supplementary Fig. S10 (a). HG002 has homozygous C alleles in the SNP site, while HG003 (father) has homozygous T alleles, and HG004 (mother) has heterozygous C/T at the same position. Lacking the C allele, the father's only haplotype in HG002 shows that the deletion is a *de novo* variation that happens to the paternal haplotype.

We choose GRCh38 rather than GRCh37 as the reference in the beta chain deletion analysis. Because the GRCh37 human genome misses the specific sequence the deletion takes place. Due to the missing segment, even HG003 and HG004 seem to have deletions in the alignment to GRCh37 (Supplementary Fig. S10 (b)). Because of the deletion in GRCh37, there are two different length insertions and one deletion reported by[37] at the site, GRCh37 chr7 position 142,494,031 (Fig S25). The first insertions is related to the sequence loss in GRCh37, so all the three members carry this insertion. The 501 bp deletion, only carried by HG002, is the HG002 deleted segment that exists in GRCh37. The genotyping indicates that this deletion is *de novo*.

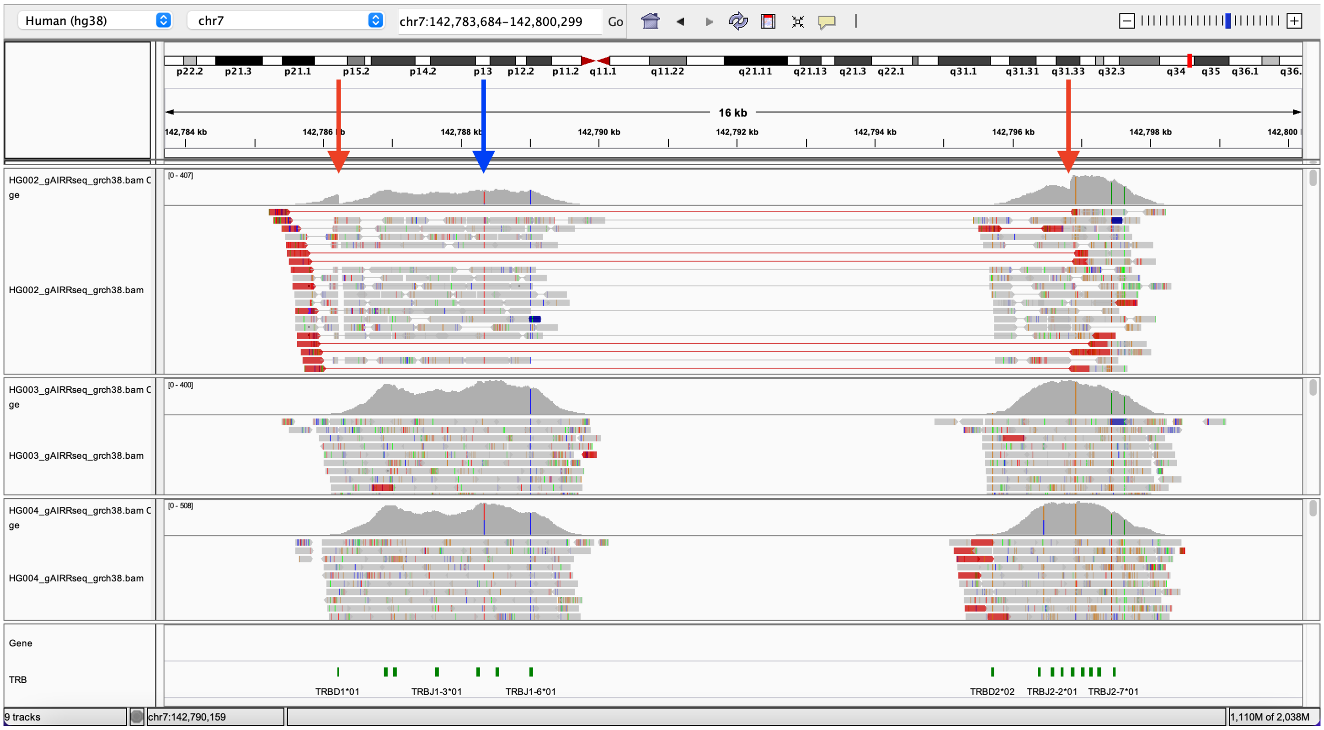

(a)

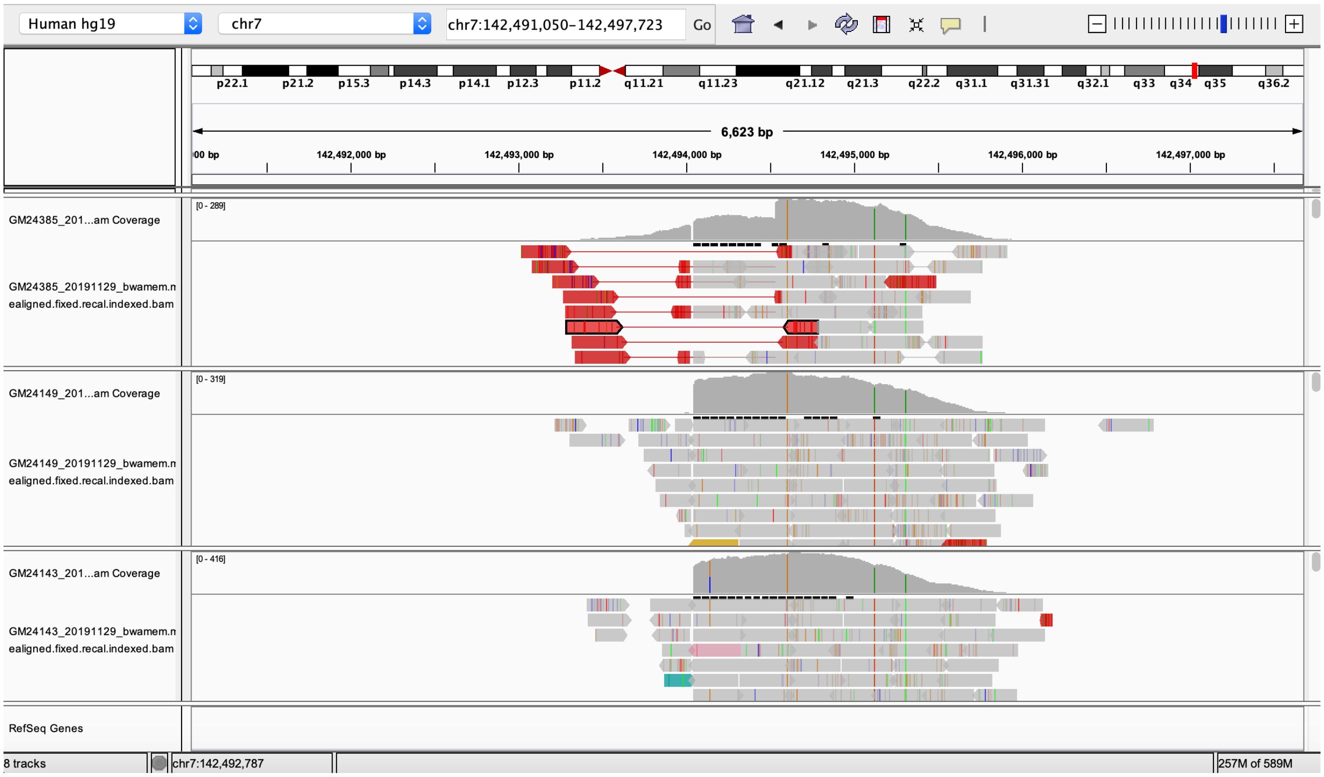

(b)

Fig. S10. The capture-based reads alignment of HG002 (son), HG003 (father), and HG004 (mother) to the GRCh38 chromosome 7 (a) and GRCh37 chromosome 7 (b)

Table S25. GIAB reports on the TRB deletion at chr7:142,494,032-142,494,532 (GRCh37)

position	ref segment	alt segment	class	HG003 genotype	HG004 genotype
chr7:142494031	18 bp	10143 bp	insertion	1/1	1/1
chr7:142494031	4543 bp	5313 bp	insertion	./.	0/0
chr7:142494032	501 bp	13 bp	deletion	0/0	0/0

14 TRB locus representation of IMGT

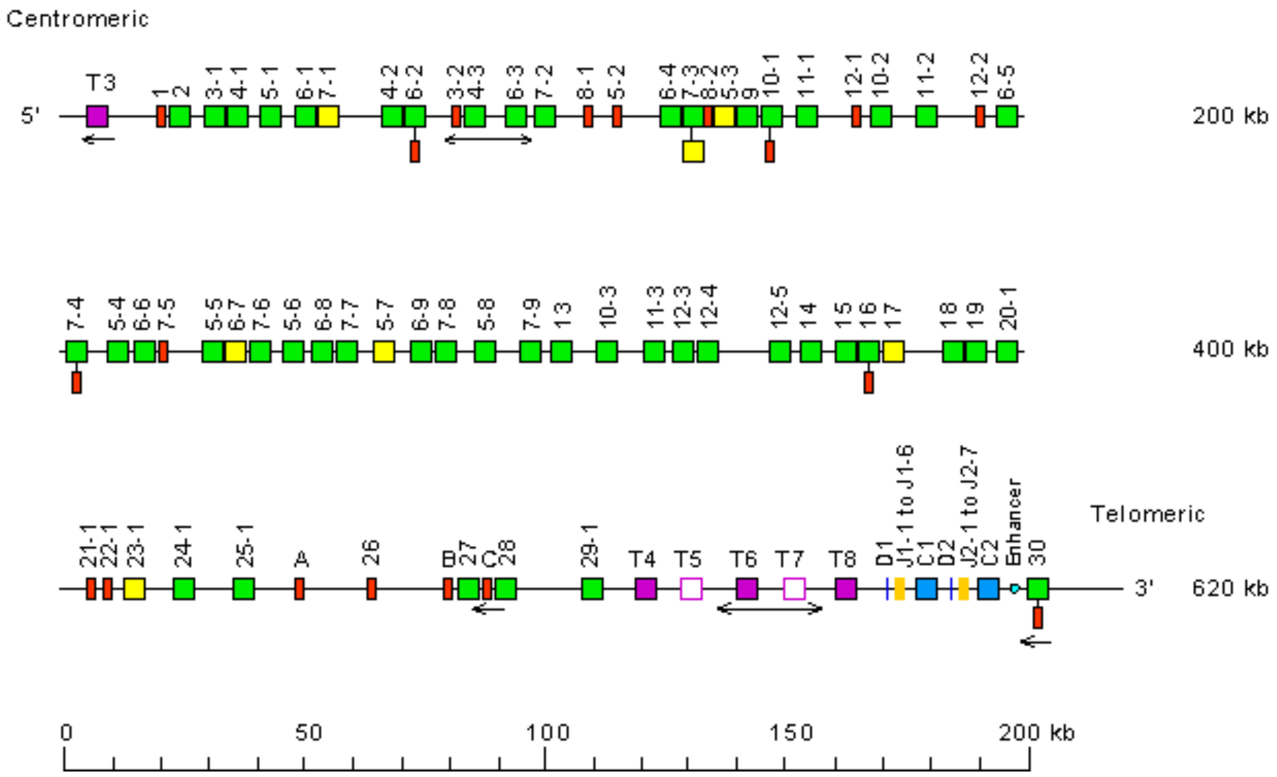

(a)

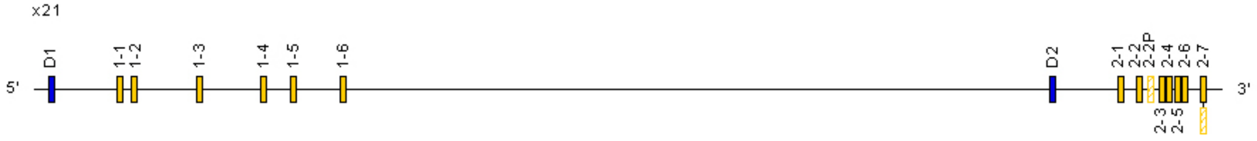

(b)

Fig. S11. The locus representation of TRB alleles (a) and details of TRBJ (b) downloaded from IMGT[38]

15 gAIRR-annotate on IG genes

Table S26. Number of IG genes gAIRR-annotated from CHM13[36], GRCh37, and GRCh38. The numbers inside the brackets indicate the number of novel alleles within the total gene numbers.

	IGH (chr14)			IGK (chr2)		IGL (chr22)		orphons	
sample	V	D	J	V	J	V	J	V	D
CHM13	132 (4)	27 (4)	9	80 (5)	5	74 (19)	7	96 (32)	10
GRCh37	116 (1)	27	9	78 (2)	5	73 (4)	7	76 (23)	10
GRCh38	123 (3)	27	9	78 (2)	5	73 (11)	7	81 (23)	10
GRCh38 chr14_KI270846v1_alt	124 (8)	27	9	N/A	N/A	N/A	N/A	N/A	N/A

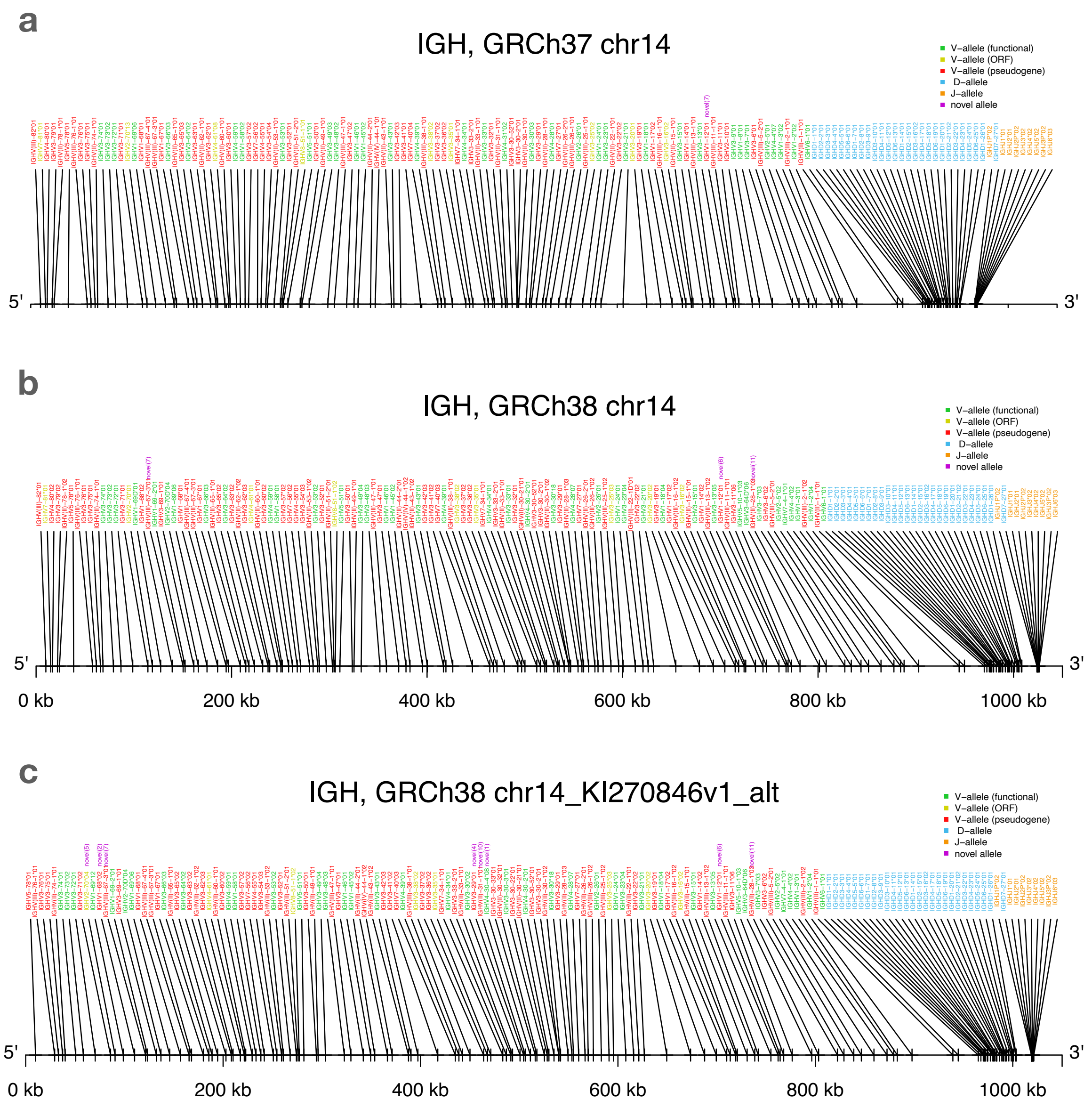

Fig. S12. gAIRR-annotate details on IGV alleles of the reference genomes GRCh37 and GRCh38.

16 gAIRR-annotate on GRCh38's alternative contig

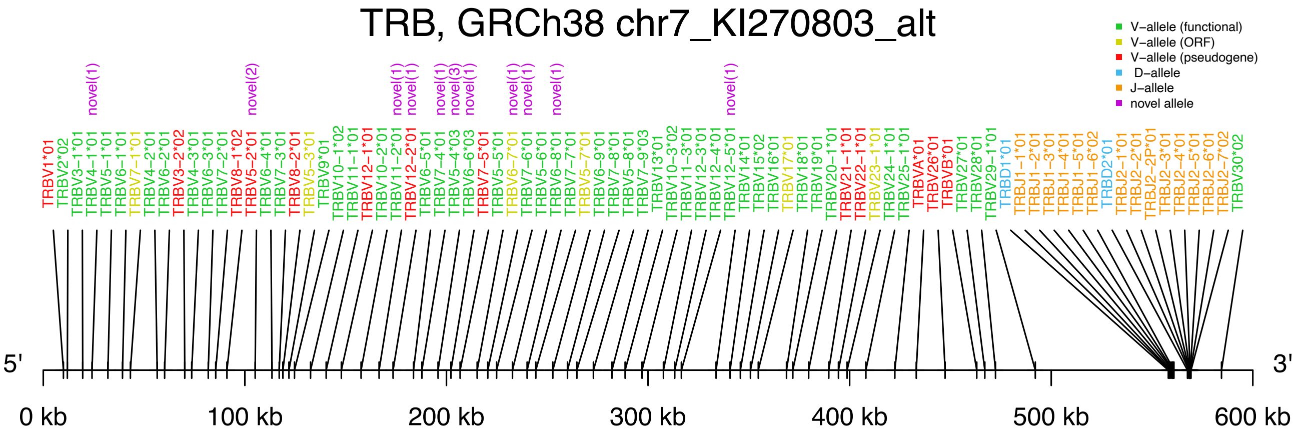

Fig. S13. The TR beta chain gAIRR-annotate representation of GRCh38 alternative contig chr7_KI270803v_alt.

17 gAIRR-seq and gAIRR-call on primary cells

Table S27. The TR alleles gAIRR-call result and gAIRR-seq result of 7 GIAB RM and a Taiwanese subject's primary cell. Since the phase information is not available in gAIRR-seq and gAIRR-call, the allele numbers include both haplotypes of the samples. The total allele number, novel allele number, and read number from EBV-trasformed cell lines and primary cells are similar.

	TRV called		TRJ called		gAIRR-seq result
sample	#novel	#total	#novel	#total	# of reads sequenced
HG001	26	186	3	86	280,579
HG002	31	188	6	89	281,494
HG003	25	171	2	86	307,312
HG004	25	176	3	89	362,271
HG005	29	166	3	86	327,354
HG006	37	189	4	90	338,361
HG007	27	174	4	90	297,186
Primary cell sample	25	174	3	86	283,691

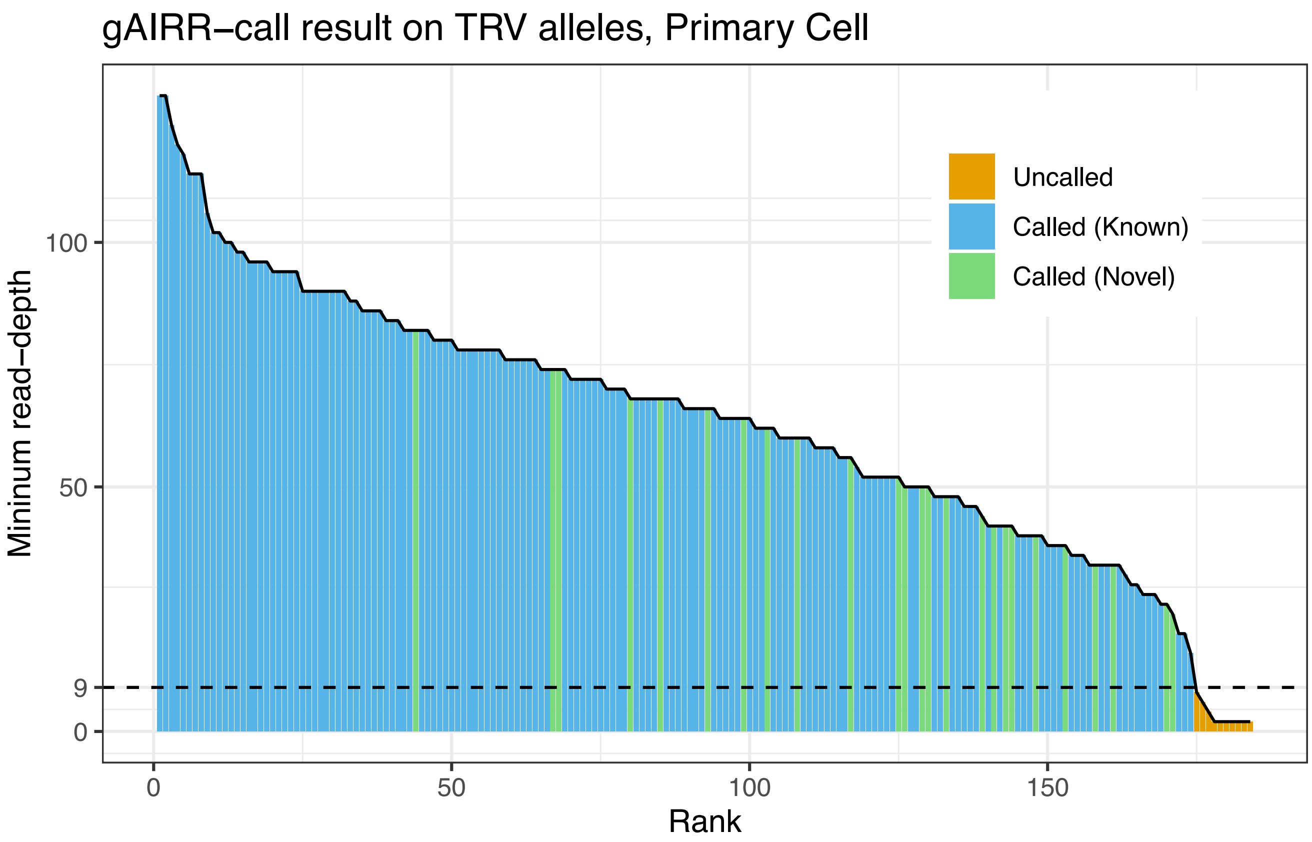

Fig. S14. gAIRR-call results on TRV alleles using PBMC data. The adaptive threshold is 9.

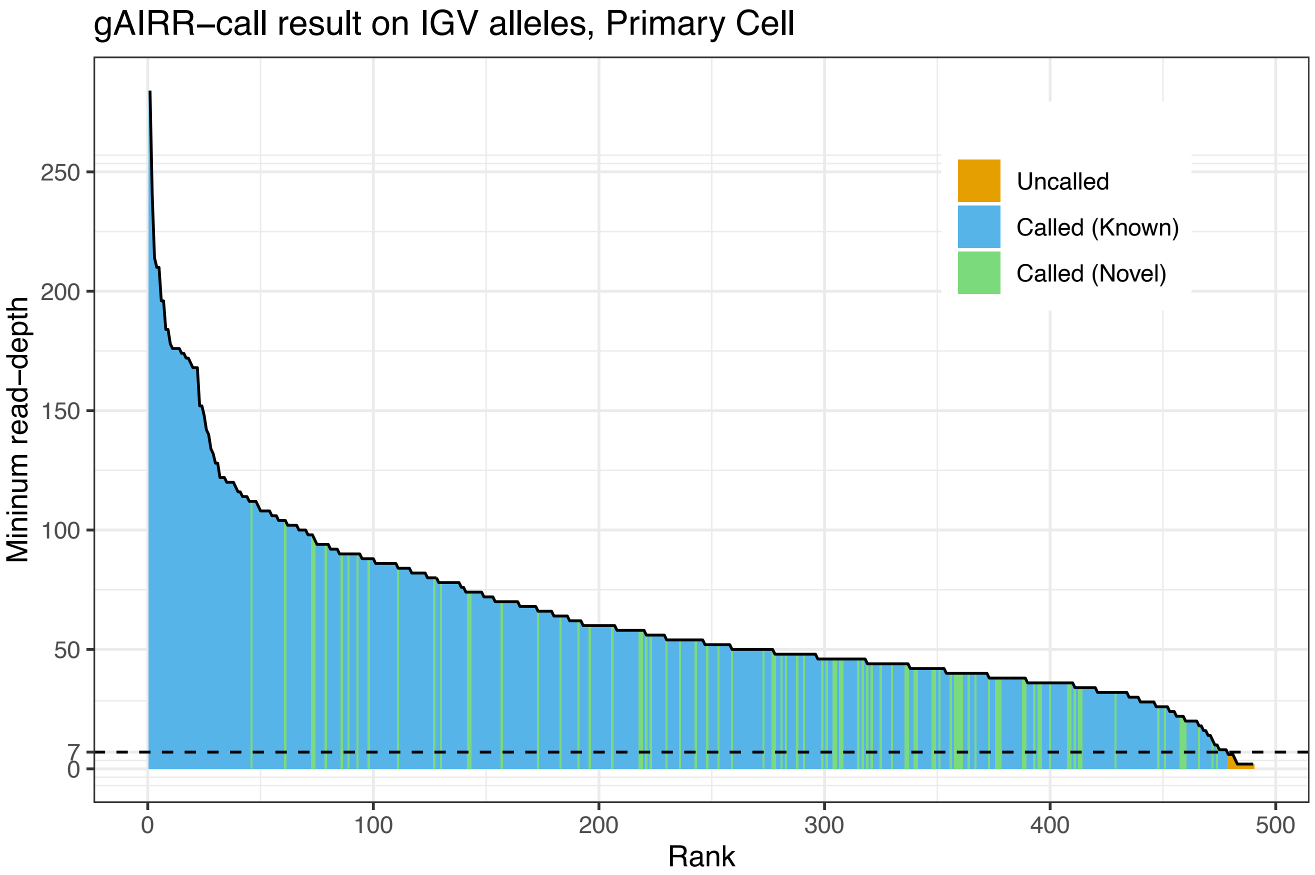

Fig. S15. gAIRR-call results on IGV alleles using PBMC data. The adaptive threshold is 7.

The settings of Supplementary Fig. S14 and Supplementary S15 are the same as Fig. 2b. However, there is no primary cell's personal assembly for verification. Thus, whether the called alleles are true positives is uncertain. Although the uncertainty, the result pattern of the primary cells is similar to that of HG001's EBV-transformed cell line in TRV alleles. In IGV alleles, the primary cell calling result is free from the impact of V(D)J recombination in EBV-transformed cell lines.

18 AIRR allele lengths in IMGT

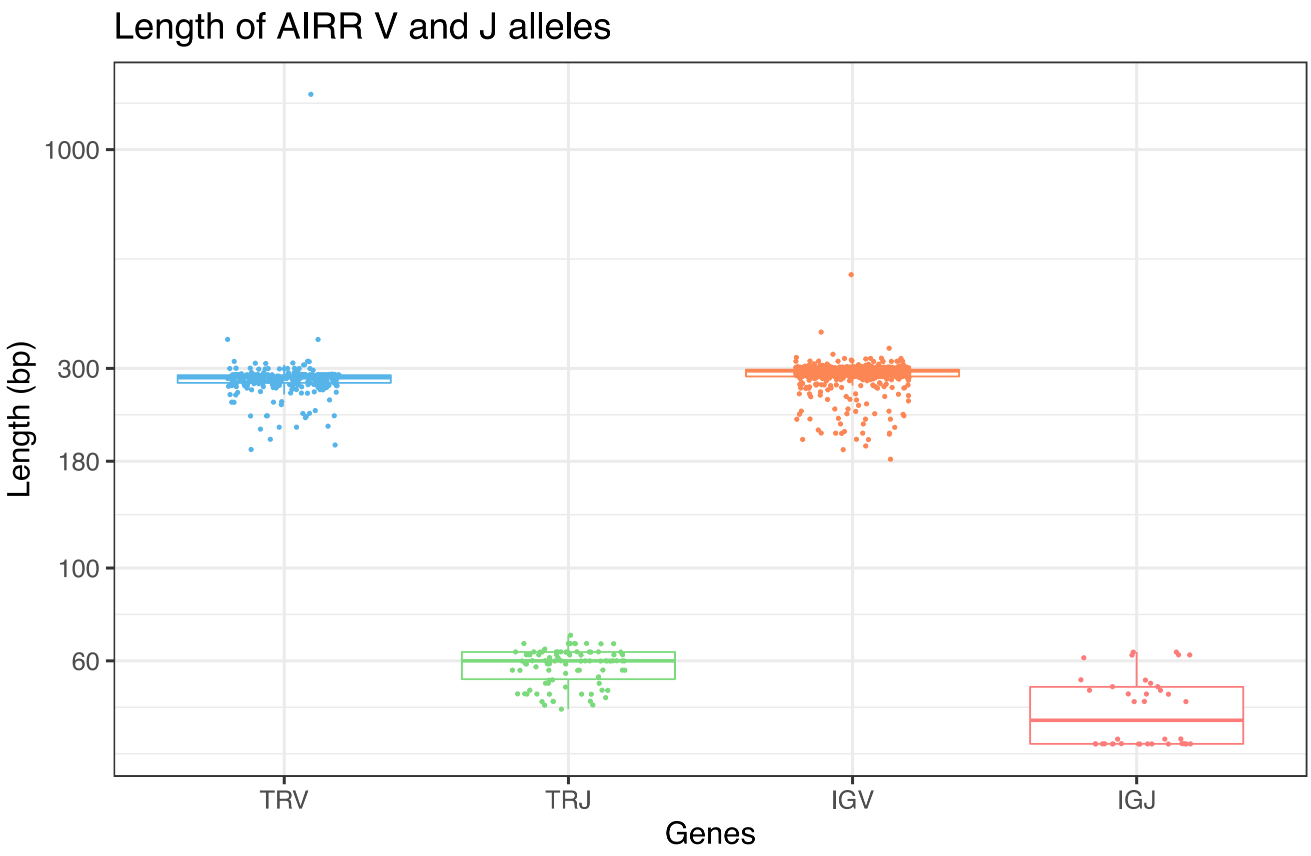

Fig. S16. The allele length distribution of AIRR V alleles and J alleles according to IMGT v3.1.22.

19 Probe design and captured-reads representation

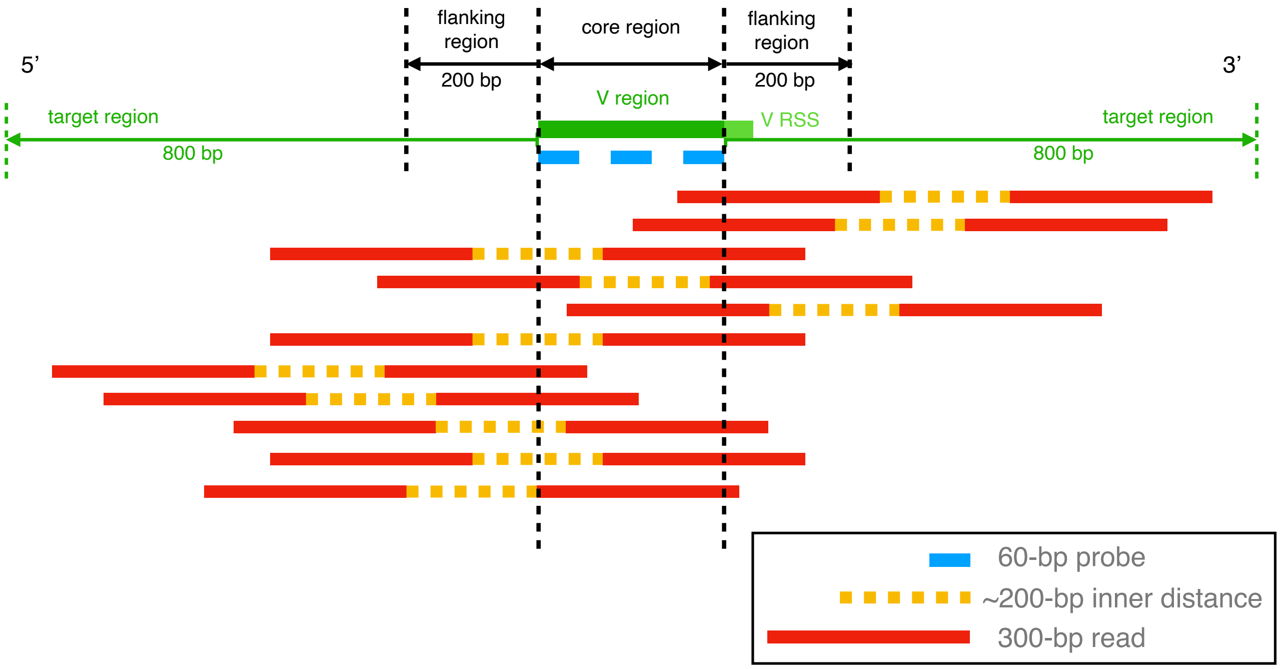

Fig. S17. The diagram representation of gAIRR-seq probes' design relative to AIRR V alleles and the captured reads.

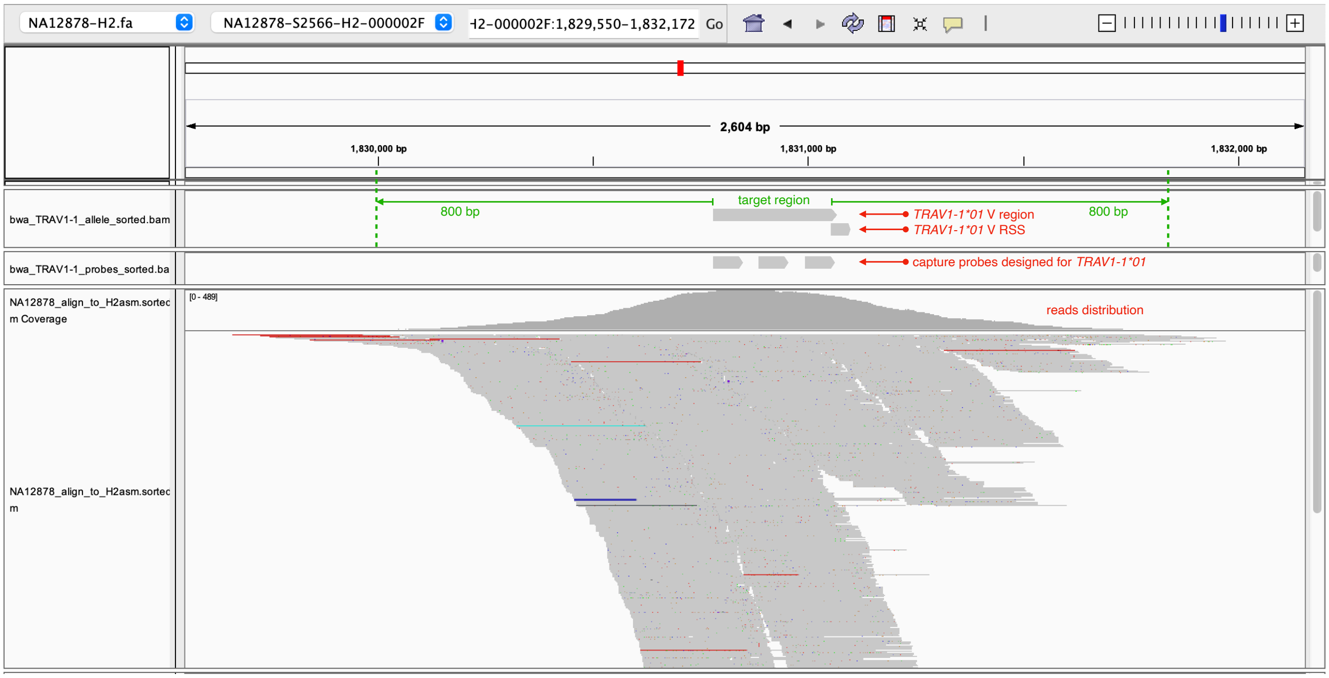

Fig. S18. The Integrated Genomics Viewer visualization of captured reads and probes aligned to HG001's gene *TRAV1-1*.

In Supplementary Fig. S17, the relative positions of V alleles, V RSS, designed gAIRR-seq probes, and the reads that can be captured are shown. We also marked the range of 200 bp flanking sequences extending from the allelic region, where extended gAIRR-call and gAIRR-annotate alleles would reach. Similarly, the 800-bp range, which we defined as the target region in gAIRR-seq's on-target rate analysis is also marked. In Supplementary Fig. S18, the V allele *TRAV1-1*01*, allele's RSS, designed probes for the allele and the captured reads of HG001 are aligned to HG001's personal assembly[31]. It can be seen that most of the reads are aligned in the target region. There is a small fraction of the reads aligned outside the target region due to the variation of fragment length.

20 gAIRR-annotate calls RSS in flanking sequences

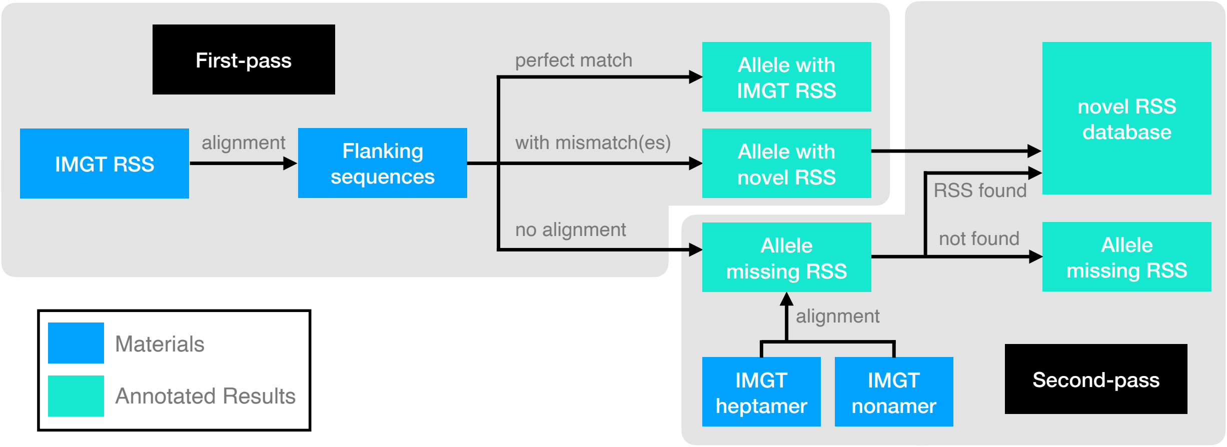

Fig. S19. The gAIRR-annotate pipeline to call the RSS in flanking sequences

In the first-pass, we align all IMGT RSS to the annotated flanking sequences. For an RSS aligned with mismatch(es), we define the aligned region as a novel RSS. The flanking sequences which cannot be aligned with IMGT RSS were re-aligned in the second-pass. In the second-pass, IMGT heptamers and nonamers are separately aligned to the flanking sequences, so the heptamer-nonamer pairs not recorded in IMGT can be found. Flanking sequences called by both gAIRR-call and gAIRR-annotate can be processed by this pipeline.

21 Annotation files of the human reference genomes

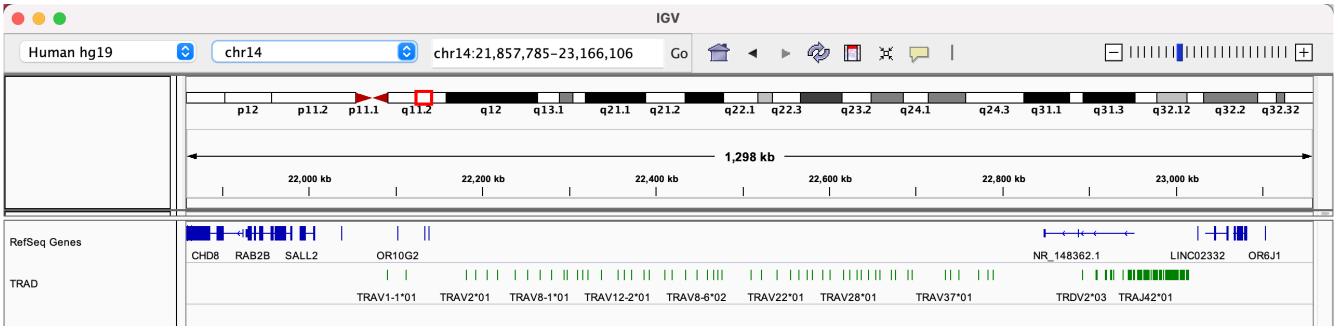

Fig. S20. Loading the TR alpha/delta chain annotation file hg19_TRAD.bed into the Integrated Genomics Viewer[45].

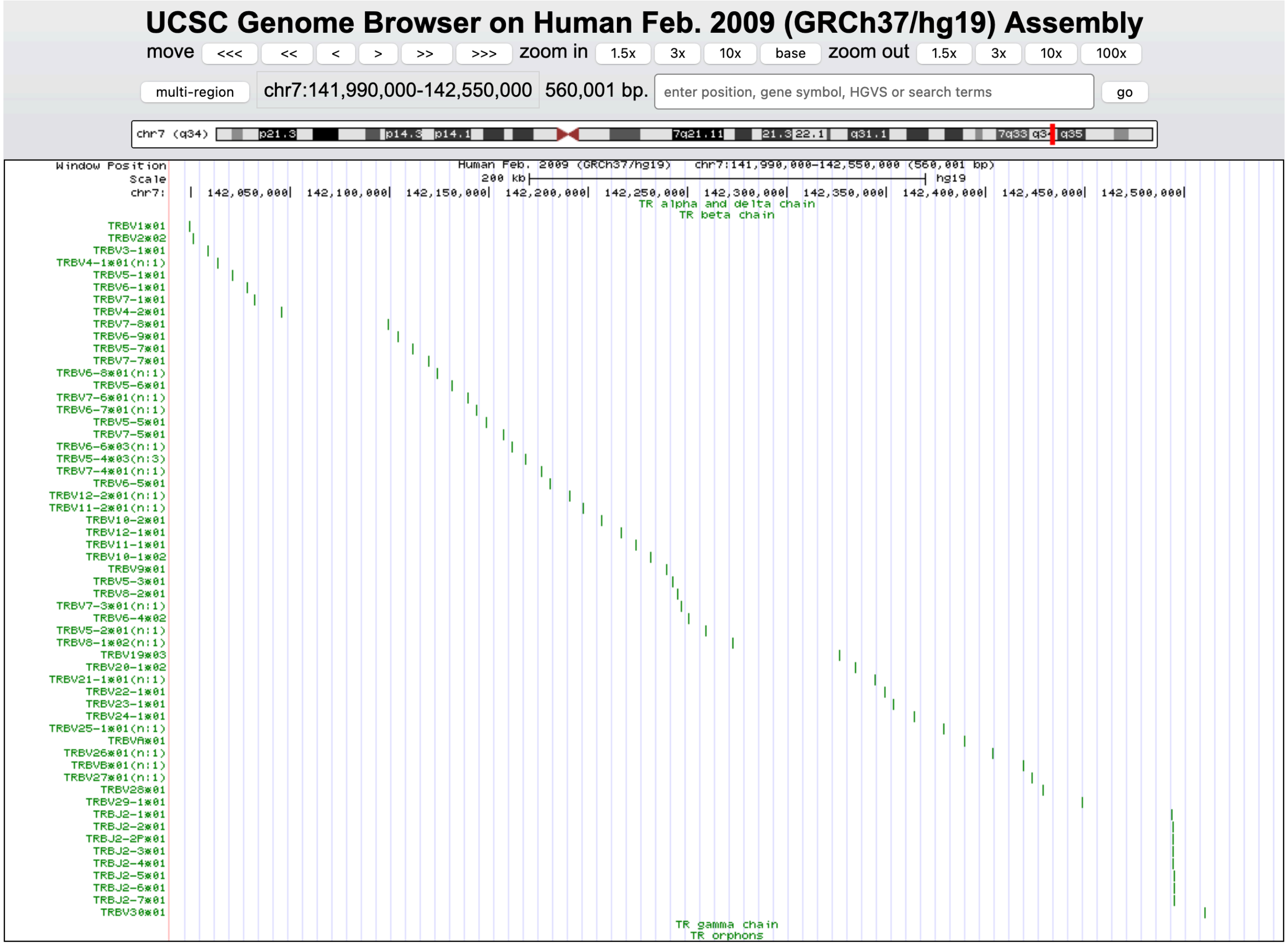

Fig. S21. Loading the TR beta chain annotation file hg19_TRB.bed into the UCSC Genome Browser[46].

Under the directory *supplementary_files/reference_genome_annotation/* in the GitHub link `https://github.com/maojanlin/gAIRRsuite` are the gAIRR-annotated bed files of the human reference genomes. In the bed files, the novel alleles will be indicated by brackets after allele names. The number after 'n:' in the brackets is the number of mismatches of the novel allele. There are four bed files recording the allele positions of GRCh37's four TR loci. Similarly, five bed files are for GRCh38's four TR loci and one locus on the alternative contig. Users can easily load the bed files into visualization tools such as Integrated Genomics Viewer[45] (Supplementary Fig. S20) or UCSC Genome Browser[46] (Supplementary Fig. S21).
